# Bayesian inference of structured latent spaces from neural population activity with the orthogonal stochastic linear mixing model

**DOI:** 10.1371/journal.pcbi.1011975

**Published:** 2024-04-26

**Authors:** Rui Meng, Kristofer E. Bouchard

**Affiliations:** 1 Biological Systems and Engineering Division, Lawrence Berkeley National Laboratory, Berkeley, California, United States of America; 2 Scientific Data Division, Lawrence Berkeley National Laboratory, Berkeley, California, United States of America; 3 Helen Wills Neuroscience Institute, University of California Berkeley, Berkeley, California, United States of America; 4 Redwood Center for Theoretical Neuroscience, University of California Berkeley, Berkeley, California, United States of America; Ernst-Strungmann-Institut, GERMANY

## Abstract

The brain produces diverse functions, from perceiving sounds to producing arm reaches, through the collective activity of populations of many neurons. Determining if and how the features of these exogenous variables (e.g., sound frequency, reach angle) are reflected in population neural activity is important for understanding how the brain operates. Often, high-dimensional neural population activity is confined to low-dimensional latent spaces. However, many current methods fail to extract latent spaces that are clearly structured by exogenous variables. This has contributed to a debate about whether or not brains should be thought of as dynamical systems or representational systems. Here, we developed a new latent process Bayesian regression framework, the orthogonal stochastic linear mixing model (OSLMM) which introduces an orthogonality constraint amongst time-varying mixture coefficients, and provide Markov chain Monte Carlo inference procedures. We demonstrate superior performance of OSLMM on latent trajectory recovery in synthetic experiments and show superior computational efficiency and prediction performance on several real-world benchmark data sets. We primarily focus on demonstrating the utility of OSLMM in two neural data sets: *μ*ECoG recordings from rat auditory cortex during presentation of pure tones and multi-single unit recordings form monkey motor cortex during complex arm reaching. We show that OSLMM achieves superior or comparable predictive accuracy of neural data and decoding of external variables (e.g., reach velocity). Most importantly, in both experimental contexts, we demonstrate that OSLMM latent trajectories directly reflect features of the sounds and reaches, demonstrating that neural dynamics are structured by neural representations. Together, these results demonstrate that OSLMM will be useful for the analysis of diverse, large-scale biological time-series datasets.

## Author summary

Extracting insight from data into the dynamic processes that produce observed phenomena is a ubiquitous challenge in biology. For example, brain functions are generated by the collective activity of many neurons. Extracting insight from biological time-series data can be challenging because the number of observations can be large, activities can be noisy, and collective dynamics can be complex. Here, we developed a new latent processes Bayesian regression model, the orthogonal stochastic linear mixing model (OSLMM) to address these challenges. Compared to competing methods, we demonstrate that OSLMM has superior recovery performance of latent trajectories in synthetic experiments and is computationally efficient. In two diverse neural data sets, we find that the latent spaces extracted from OSLMM are directly structured by task parameters. Our results demonstrate that latent dynamics can be structured by representations and suggest that OSLMM will be useful for data-driven discovery in large-scale biological time-series data.

## Introduction

Complex brain functions are the result of the activity of large populations of neurons [1]. From perceiving sounds to producing arm movements, a major focus of modern neuroscience is to record and understand the structure of latent spaces/dynamics of neural population activity [2]. Powered in large part by recent technological advancements, the number of simultaneously recorded neurons has been growing rapidly. However, extracting understanding from such data sets remains an open analysis challenge because the number of simultaneously recorded neural signals can be large, the collective dynamics of neural population activity can be complex, and there can be substantial variability across individual trials. Neuroscientists often draw conclusions about brain function from the organization (or lack thereof) of latent population activity with respect to exogenous variables (e.g., sound frequency, reach angle) [3–5]. For example, latent neural dynamics that lack organization according to reach kinematics has been interpreted as evidence that motor cortex should be viewed as a dynamical system, not a representational system [3]. However, lack of structured latent spaces could reflect methodological short-comings, not necessarily the latent structure in the data. Thus, it is important that analysis methods for latent spaces of population neural activity are interpretable while imposing as little structure as possible so as to let the data “speak for itself”.

The latent structure of population neural activity can result from both internal processing and exogenous factors. A common finding is that, while the ambient dimension (e.g., number of neurons) can be large, the intrinsic dimensionality of population activity are often low (i.e., they are often confined to a lower dimensional subspace) [2]. Furthermore, neural activity across multiple trials of the same exogenous variable can be different, as illustrated in a diversity of experiments [6]. Therefore, it is becoming increasingly important to analyze neural population activity across multiple trials directly, without first averaging [2, 7–9]. Finally, it has been observed that there are time-dependent changes in correlation structure that are temporally aligned to exogenous variables (e.g. [10, 11]). Thus, it is important for methods to be able to handle non-stationary correlations. However, methods that address all these challenges are nascent.

Different methodological approaches have been developed to extract low-dimensional latent structure from single-trial population neural data. Generally speaking, such methods model two mapping functions: the mapping function between time and latent trajectories and the mapping function from latent trajectories to neural observations. One of the most popular approaches is Gaussian Process Factor Analysis (GPFA) proposed by [2]. It models the neuron responses as a linear mapping of latent trajectories and the mapping between the latent trajectory and time is modeled by independent Gaussian processes (GP). The linear mapping models the correlation of neurons and GPs provide a flexible way to model latent trajectories. Moreover the GPs also impose smoothness into latent trajectories which is important for analysis of noisy single-trial data and aids visual interpretation. However, GPFA assumes time invariant correlations, which is known to not hold in real neural data [6, 10, 11]. Broadly speaking, GPFA belongs to the class of linear models of coregionalization [12, 13]. Several methods built around this class of models are capable of handling nonstationary covariances [14, 15], datasets with large numbers of samples, and high-dimensional datasets [16, 17]. More recently, like GPFA, [8, 18] use the linear coupling between latent dynamics and neural responses but model the dynamics using linear dynamic systems and recurrent networks, respectively. On the other hand, several studies have introduced nonlinear coupling between latent dynamics and neural responses, such as Gaussian processes [7, 9] and neural networks [19]. Although those nonlinear mappings are flexible, they impede geometric interpretation of the extracted latent spaces.

Here, we developed a new, general Bayesian regression framework for high-dimensional time series data with latent dynamics, the Orthogonal Stochastic Linear Mixing Model (OSLMM). We first developed the Stochastic Linear Mixing Model (SLMM), where we employ Gaussian processes for dynamics and an adaptive linear function to model the coupling relation between latent dynamics and neural responses. OSLMM then puts an orthogonality constraint on the adaptive coefficient matrices of the SLMM. We derived theoretical computational benefits of OSLMM which are confirmed by empirical results in real datasets. Compared with GPFA, we demonstrated that OSLMM had better latent dynamics recovery in synthetic experiments utilizing the Lorenz system and has superior predictive performance on real benchmark datasets and neural data. Most importantly, OSLMM extracted insightful latent subspaces in application to diverse population neurophysiology recordings. Specifically, in *μ*ECoG recordings from rat auditory cortex, OSLMM subspaces exhibited monotonic structure of stimulus amplitude and frequency. In simultaneous recordings of multiple single-units from motor cortex of monkeys performing reaches, OSLMM subspaces were structured by reach angle and speed. The functional organization observed in the OSLMM extracted subspaces matches expectations from the response properties of individual neurons. These results imply that dynamical systems and representational systems perspectives on brain computations are not as incompatible as previously proposed. Together, these results demonstrate that OLSMM extracts latent structure from time-series data that provide insight into the neurobiological processes that generated observed data.

## Materials and methods

We first describe the stochastic linear mixing model and orthogonal stochastic linear mixing model as well as their inference approaches. Both models assume zero mean, and so we have a centering step if the data is not zero mean. Then we describe the synthetic data generating process for the Lorenz system for model validation. Finally, we provide details of analysis and evaluation metrics on the neural data.

### Stochastic linear mixing model

Let ***f***(⋅) = [*f*_1_(⋅), …, *f*_*Q*_(⋅)] be a vector-valued function composed of *Q* independent latent functions. Each latent function is independently sampled from a GP prior with a squared exponential covariance function such that $f_{q}∼GP\left(0,k_{f}\right)$ with *k*_*f*_(t, t) = 1. *W*(t) is a *P* × *Q* input dependent coefficient matrix and Σ is a *P* × *P* covariance matrix of observational noise, where P is the number of time-varying mixing processes. SLMM models the output function as a linear combination of latent functions corrupted with observation noises. We call ***f*** the latent processes and *W* the mixing coefficient processes. Specifically, the SLMM is given by the following generative model:

$$\begin{array}{cc} f_{q} & ∼GP(0,k_{f_{q}})\;,\;\text{latent}\;\text{processes}\\ g(t)|W(t),f(t) & =W(t)f(t)\;,\;\text{mixing}\;\text{mechanism}\\ y(t)|g(t) & ∼N(g(t),\Sigma )\;.\;\text{noise}\;\text{model} \end{array}$$

The SLMM is a generalization of the instantaneous linear mixing model [20]. Instead of employing deterministic mixing coefficients **W**, the SLMM explicitly assumes that it depends on time t. This mixing mechanism with independent latent processes is called the spatially varying linear model of corregionalization [14] in the spatial statistics literature. Recently, [15] proposed a general regression framework based on this mixing mechanism and demonstrated successful analysis of electronic health records. On the other hand, replacing latent processes ***f***(t) with noisy latent processes ***f***(t) + *σ*_*f*_***ϵ***, assuming homogeneous noise such that $\Sigma =\sigma_{y}^{2}I_{P}$ and modeling each element of *W*(t) via a Gaussian process takes the SLMM to be the exact Gaussian process regression network (GPRN) in [21].

Following [21], we assume all the latent functions share the same covariance function *k*_*f*_, and also assume that each mixing coefficient *w*_*ij*_(t) is independently sampled from a GP prior with the same covariance function *k*_*w*_. We denote the values of *f*_*q*_ at times **T** = [t_1_, …, t_*T*_]′ by **f**_*q*,⋅_ = [*f*_*q*_(t_1_), …, *f*_*q*_(t_*T*_)]′, the values of *w*_*ij*_ at times **T** by **w**_*ij*_ = [*w*_*ij*_(t_1_), …, *w*_*ij*_(t_*T*_)]′. The joint probability of observed outputs **Y** = [**y**_1_, …, **y**_*T*_] with **y**_*t*_ = ***y***(t_*t*_), and latent variables **W** = [*W*(t_1_), …, *W*(t_*T*_)] and latent functions **F** = [***f***(t_1_), …, ***f***(t_*T*_)] is

$$\begin{array}{c} p\left(Y,W,F|X,\theta_{f},\theta_{w},\Sigma \right)=\prod_{t=1}^{T}N\left(y_{t}|W_{t}f_{t},\Sigma \right)\prod_{i=1}^{P}\prod_{j=1}^{Q}N\left(w_{ij}|0,K_{w}\right)\prod_{q=1}^{Q}N\left(f_{q,\cdot }|0,K_{f}\right) \end{array}$$ (1)

where **W**_*t*_ is a *P* × *Q* coefficient matrix at time t_*t*_ in which [**W**_*t*_]_*ij*_ = *w*_*ij*_(t_*t*_), **f**_*t*_ = ***f***(t_*t*_). **K**_*w*_ and **K**_*f*_ are the covariance matrices estimated at times **T**, and model parameters are Θ = (*θ*_*f*_, *θ*_*w*_, Σ). The hyper-parameters of Gaussian processes corresponding to *f* and *w* include (amplitude) scale and length scale parameters. We have the detailed explanation in Appendix A in S1 File for SLMM method and in Appendix C in S1 File for OSLMM method.

Learning in the SLMM is equivalent to inference of the posterior distribution of latent variables and model parameters. Latent variables consist of mixing coefficients **W** and latent functions **F**, and model parameters include the covariance matrix of observation noise Σ and hyper-parameters in GPs. The most computationally expensive component of the learning procedure comes from inference of latent variables. We note that the conditional posterior of mixing coefficients *p*(**W**|**F**, **Y**, **T**, *θ*_*f*_, *θ*_*w*_, Σ) and the conditional posterior of latent functions *p*(**F**|**W**, **Y**, **T**, *θ*_*f*_, *θ*_*w*_, Σ) have close-form multivariate Gaussian distributions with dimension *PQT* and *QT*. The complexity of learning them are $O(P^{3}Q^{3}T^{3})$ and $O(Q^{3}T^{3})$ respectively. Hence, Gibbs sampling for **W** and **F** would be difficult for large datasets. [21] propose a Markov-chain Monte-Carlo (MCMC) approach to jointly sample them via elliptical slice sampling (ESS), an acceptance-rejection sampling method [22]. The time complexity of ESS depends on computing the joint distribution of Eq (1) which takes $O(PQT^{3})$ (shown in the supplementary of [21]). Although in principle ESS relieves the computational burden, ESS still does not work for large datasets in practice because of poor mixing.

Our inference conditionally samples latent variables **W** and **F** given model parameters Θ via ESS and conditionally samples Θ given **W** and **F**. The details of sampling model parameters are described in Appendix A in S1 File. Similar to the inference in [21], our inference is not efficient, because ESS suffers from low efficiency and slow time to convergence. Therefore, we next propose a new regression framework, the orthogonal stochastic linear mixing model that introduces an orthogonality constraint amongst the mixing coefficients and significantly improves the inference efficiency theoretically and empirically.

### Orthogonal stochastic linear mixing model

In SLMM, the most burdensome computation comes from the inference of mixing coefficients **W**, which includes *PQT* model parameters. To improve the inference efficiency, we simplify the model by introducing an orthogonality constraint amongst the mixing coefficients. We call this new model the orthogonal stochastic linear mixing model (OSLMM).

Instead of explicitly modeling the mixing coefficient processes *W* via GPs, OSLMM takes the eigen-decomposition of the variance-covariance matrix of the latent signal ***g***(t) given *W*(t), implying that var (***g***(t)) = *W*(t)*W*(t)′ = *U*(t)*S*(t)*U*(t)′, where the columns of $U\left(t\right)\in R^{P\times Q}$ are orthonormal and $S\left(t\right)\in R^{Q\times Q}$ is a positive diagonal matrix. Then *W*(t) can be decomposed as $W\left(t\right)=U\left(t\right)S^{\frac{1}{2}}\left(t\right)$. We simplify the structure of mixing coefficients by assuming *U*(t) is independent from input t: $W\left(t\right)=US^{\frac{1}{2}}\left(t\right)$. Then the latent signal ***g***(*t*) stays in the subspace spanned by the orthonormal basis of **U**. This assumption is in accordance with the observation that high-dimensional data usually lie on a low-dimensional manifold in many real-world problems [4].

**Fig 1:**
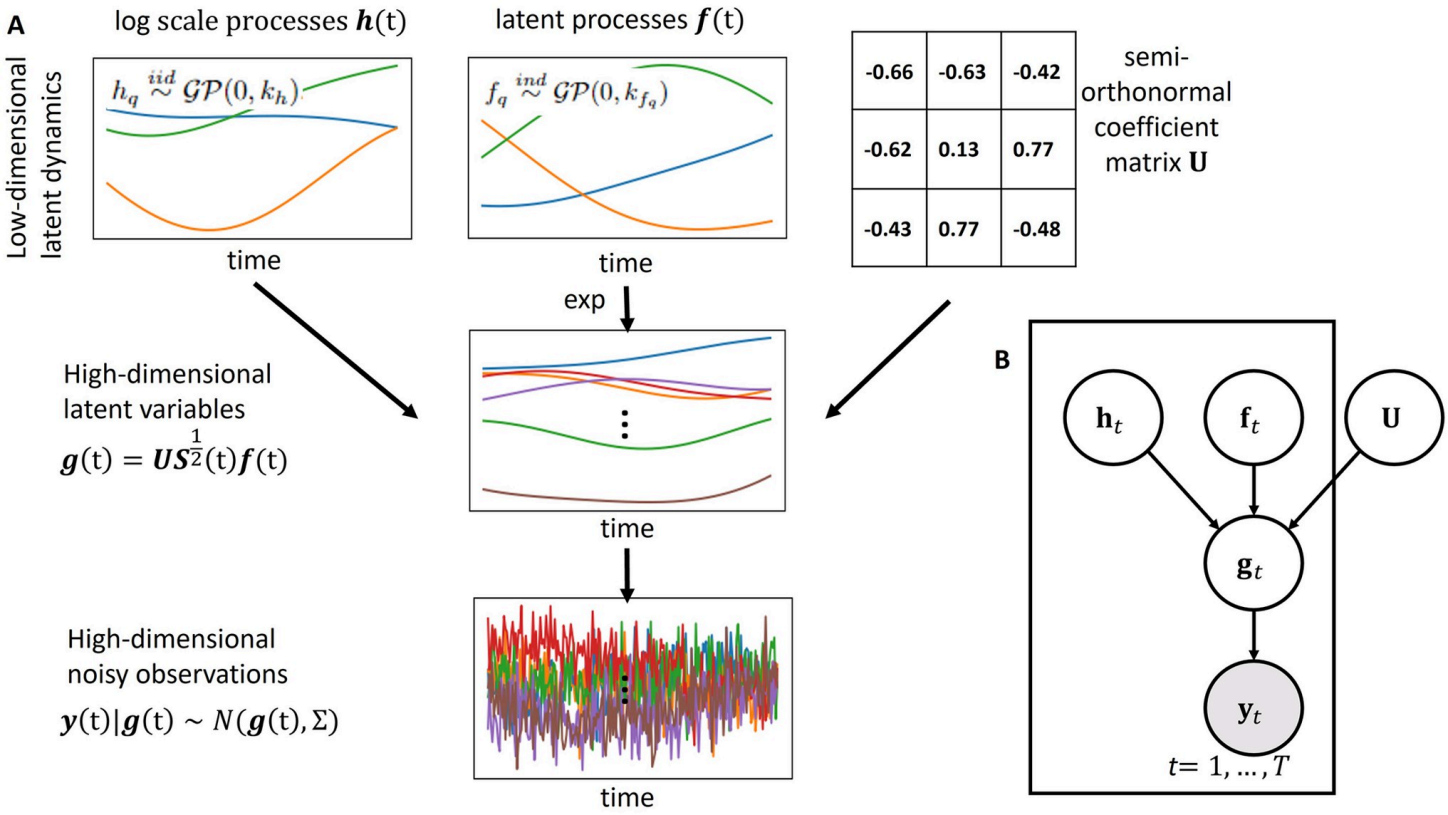
Schematic diagram of the Orthogonal Stochastic Linear Mixing Model (OSLMM). (A) illustrates data generated by the model with a three-dimensional latent processes. Note that $S^{\frac{1}{2}}\left(t\right)$ is a positive diagonal matrix with log([*S*^1/2^(t)]_*qq*_) = *h*_*q*_(t). (B) illustration of the graphical model of OSLMM.

The orthogonal stochastic linear mixing model (OSLMM) is an SLMM where the latent signal ***g***(t) is expressed as $g\left(t\right)=W\left(t\right)f\left(t\right)=US^{\frac{1}{2}}\left(t\right)f\left(t\right)$ where **U** is a *P* × *Q* matrix with orthonormal columns, *S*(t) is a *Q* × *Q* positive diagonal matrix indexed by input t, and $\Sigma =\sigma_{y}^{2}I$. In order to model the positive diagonal matrix function $S^{\frac{1}{2}}\left(t\right)$, OSLMM assumes that each element on the diagonal of $S^{\frac{1}{2}}\left(t\right)$ is in the logarithmic scale, $h_{q}\left(t\right)=\text{log}\left({\left[S^{\frac{1}{2}}\left(t\right)\right]}_{qq}\right)$, with a GP prior modeled by a squared exponential covariance function such that $h_{q}\overset{iid}{∼}GP\left(0,k_{h}\right)$. We denote the values of *h*_*q*_(*t*) at times **T** as **H** = [**h**_1_, …, **h**_*T*_] where ***h***_*t*_ = [*h*_1_(t_1_), …, *h*_*Q*_(t_*T*_)]′. We display the schematic diagram of OSLMM with the latent dimension size *Q* = 3 in Fig 1 as well as the corresponding graphical model. In comparison with SLMM, the number of latent variables of OSLMM is reduced from *PQT* + *QT* to *PQ* + 2*QT*. In practice, this reduction in parameters renders inference possible for large datasets. In addition, we develop an efficient inference framework via sufficient statistics as follows.

Similar to [20], for each time stamp t_*t*_, we first propose the projection matrix $R_{t}=S_{t}^{-\frac{1}{2}}U^{\prime }$, where **S**_*t*_ = *S*(t_*t*_). We prove that conditional on **U** and **S**_*t*_, **R**_*t*_**y**_*t*_ is a maximum likelihood estimate for **f**_*t*_. In addition, **R**_*t*_**y**_*t*_ is a minimally sufficient statistic for **f**_*t*_. The detailed proofs are provided in Appendix B in S1 File. Those summary statistics lead to the fact that for any prior *p*(**f**_*t*_) over **f**_*t*_, we have

$$\begin{array}{c} p\left(f_{t}|y_{t}\right)=p\left(f_{t}|R_{t}y_{t}\right)\;,\;R_{t}y_{t}|f_{t}\overset{ind}{∼}N\left(R_{t}y_{t}|f_{t},\Sigma_{R_{t}}\right) \end{array}$$ (2)

where $\Sigma_{R_{t}}=S_{t}^{-\frac{1}{2}}U^{\prime }\Sigma US_{t}^{-\frac{1}{2}}$. It suggests the posterior of **f**_*t*_ depends only on low dimensional summary statistics **R**_*t*_**y**_*t*_. Moreover, when Σ has the form $\Sigma =UD_{1}U^{\prime }+\sigma_{y}^{2}I$, the variance-covariance matrix is a diagonal such that $\Sigma_{R_{t}}=S_{t}^{-\frac{1}{2}}D_{1}S_{t}^{-\frac{1}{2}}+\sigma_{y}^{2}S_{t}^{-1}$. This leads to a linear learning complexity with respect to latent functions **f** in Eq (3) (see below). In the rest of this work, we assume a homogeneous noise $\Sigma =\sigma_{y}^{2}I$, and thus $\left\{\Sigma_{R_{t}}\right\}$ are diagonal.

We refer to $c\left(t\right)=S^{\frac{1}{2}}\left(t\right)f\left(t\right)$ as the orthonormalized latent functions. The orthonormality refers to U, and c(t) is the coefficient functions in terms of the orthonormal basis U. Each dimension of ***c***(t) represents a scaled ***f***(t) at each input t. Similar to the orthonormalized neural state in GPFA [2], the orthonormalized latent functions can explain the amount of data covariance. Since we put the orthogonality constraint on U during the training, we do not need to do a final SVD on c(t) as is done in GPFA. Also, similar to the spirit of PCA [1, 23], this orthonormality constraint penalizes redundancy in latent representations [24] and thus contributes to a better low-dimensional visualizations of the latent structure. We also note that the GPFA impose the orthogonality after inference, which may lead to mixing of data effects in latent factors that can not demixed by post-hoc orthogonalization in an unsupervised manner. In OSLMM, this orthogonality constraint is imposed directly during inference, allowing it to capture orthogonal structure in the data directly. This contributes to better interpretation of latent trajectories.

As for inference, we propose a Markov chain Monte Carlo (MCMC) algorithm for OSLMM via Gibbs sampling, which updates latent functions and model parameters iteratively from their conditional posterior distributions. First, because of Eq 2, the conditional posterior of latent functions **F** is

$$\begin{array}{cc} p(F|H,U,Y,X,\theta_{f},\theta_{w},\Sigma ) & \propto \prod_{t=1}^{T}N\left(R_{t}y_{t}|f_{t},\Sigma_{R_{t}}\right)\prod_{q=1}^{Q}N\left(f_{q,\cdot }|0,K_{f}\right)\\ & =\prod_{q=1}^{Q}N\left(f_{q,\cdot }|{\left(K_{f}^{-1}+{\tilde{\Sigma }}_{q}^{-1}\right)}^{-1}\left({\tilde{\Sigma }}_{q}^{-1}{\tilde{y}}_{q}\right),{\left(K_{f}^{-1}+{\tilde{\Sigma }}_{q}^{-1}\right)}^{-1}\right)\;, \end{array}$$ (3)

where ${\tilde{\Sigma }}_{q}=\text{diag}\left({\left[\Sigma_{R_{1}}\right]}_{qq},\ldots ,{\left[\Sigma_{R_{T}}\right]}_{qq}\right)$ and ${\tilde{y}}_{q}={\left({\left[R_{1}y_{1}\right]}_{q},\ldots ,{\left[R_{T}y_{T}\right]}_{q}\right)}^{\prime }$.

Because this conditional posterior can be factorized into the product of each latent dimension *q*, and each conditional posterior is a multivariate Gaussian distribution, the learning complexity is $O(T^{3}Q)$, linear to the latent dimension size *Q*. The conditional posterior of **H**, is

$$\begin{array}{cc} p(H|F,U,Y,X,\theta_{f},\theta_{w},\Sigma ) & \propto \prod_{t=1}^{T}N\left(y_{t}|US_{t}^{\frac{1}{2}}f_{t},\Sigma \right)\prod_{q=1}^{Q}N\left(h_{q,\cdot }|0,K_{h}\right)\\ & \propto \prod_{t=1}^{T}\text{exp}\left(-\frac{1}{2}{\left(y_{t}-US_{t}^{\frac{1}{2}}f_{t}\right)}^{\prime }\Sigma^{-1}\left(y_{t}-US_{t}^{\frac{1}{2}}f_{t}\right)\right)\prod_{q=1}^{Q}N\left(h_{q,\cdot }|0,K_{h}\right)\;, \end{array}$$ (4)

where **h**_*q*,⋅_ = (*h*_*q*_(t_1_), …, *h*_*q*_(t_*T*_))′.

As Σ is diagonal, this likelihood can be factorized for each time index *t* and each output dimension *p*, so the computational complexity of this posterior is $O(\text{max}\left(PT,T^{3}\right))$. Since the closed-form expression of each posterior is intractable, we sample them via the elliptical slice sampling [22].

To sample **U**, because **U** is on the Stiefel manifold where the columns are orthonormal, we parametrize **U** with the polar decomposition such that $U\overset{d}{=}U_{V}=V{\left(V^{T}V\right)}^{-\frac{1}{2}}$ [25], where $V\in R^{P\times Q}$ is a random matrix. We assume *p*_**U**_(**U**) is uniform and thus **V** follows a matrix angular central Gaussian distribution, MACG(**I**_*P*_), corresponding to $V∼N_{P,Q}\left(0,I_{P},I_{Q}\right)$ [26]. Hence, the conditional posterior of **V** is

$$\begin{array}{cc} p(V|F,H,Y,X,\theta_{f},\theta_{w},\Sigma ) & \propto \prod_{t=1}^{T}N\left(y_{t}|US_{t}^{\frac{1}{2}}f_{t},\Sigma \right)N_{P,Q}\left(V|0,I_{P},I_{Q}\right)\\ & \propto \prod_{t=1}^{T}\text{exp}\left(-\frac{1}{2}{\left(y_{t}-US_{t}^{\frac{1}{2}}f_{t}\right)}^{\prime }\Sigma^{-1}\left(y_{t}-US_{t}^{\frac{1}{2}}f_{t}\right)\right)N_{P,Q}\left(V|0,I_{P},I_{Q}\right)\;. \end{array}$$ (5)

We sample **V** via elliptical slice sampling and the computational complexity of this posterior is O(max(PT,PQ)).

**Fig 2:**
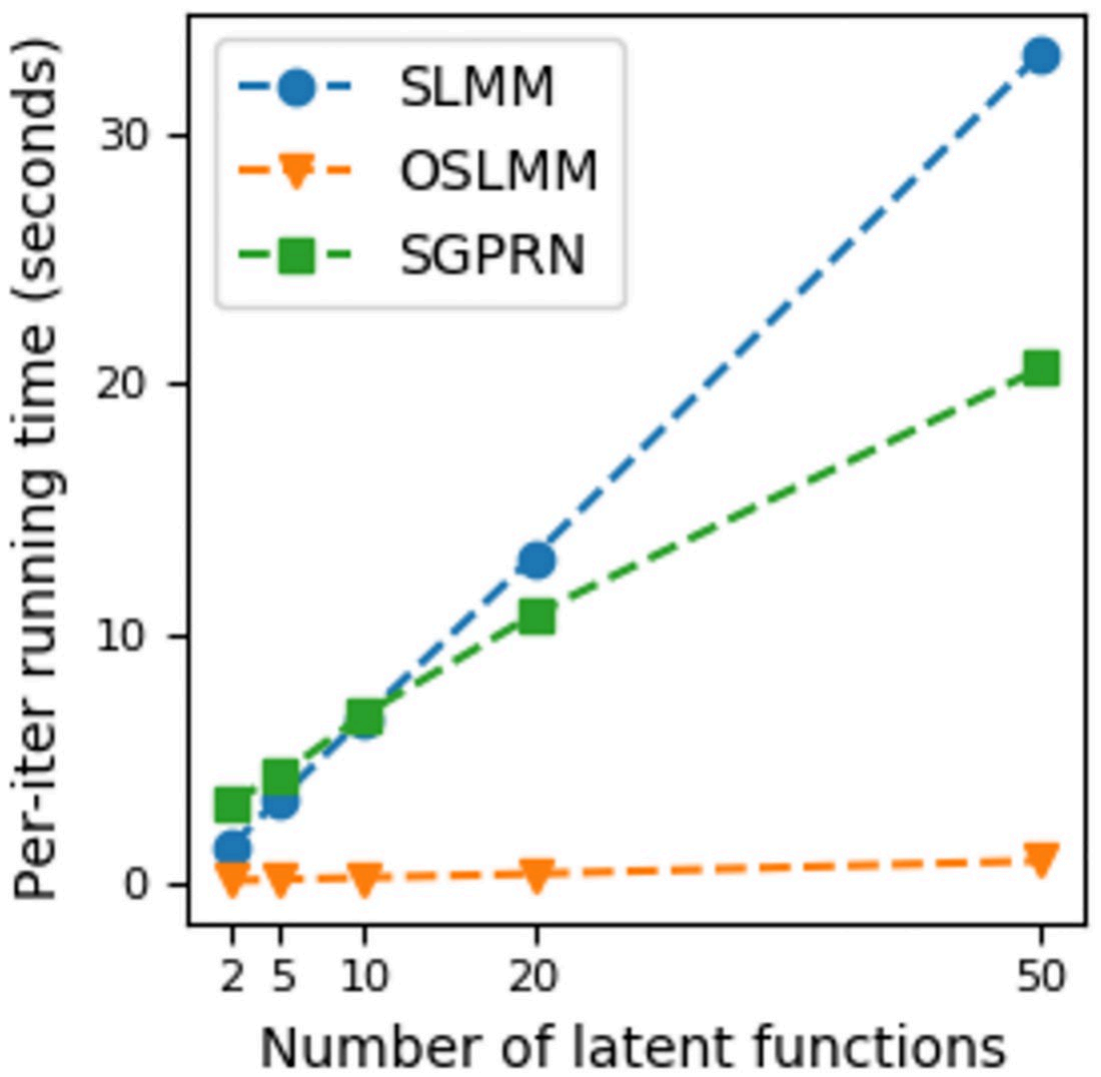
Training OSLMM on neural data scales well with the number of latent functions. The running time per iteration with different number of latent functions for the Stochastic Linear Mixing Model (SLMM), the Orthogonal Stochatic Linear Mixing Model (OSLMM), and the Stochastic Gaussian Process Regression Network (SGPRN).

Finally, to update model parameters Θ, we employ the Metropolis-Hastings method and the details are discussed in Appendix C in S1 File. This inference takes $O(\text{max}\left(QT^{3},PT,PQ\right))$ time, which is linear in the number of latent dimensions *Q* and output variable dimensionality *P*. Empirically, we compare the training speed of OSLMM to that of SLMM and sparse Gaussian process regression network (SGPRN) [27] in a neural dataset (*μ*ECoG recordings from auditory cortex) with output dimension 128 [28]. This experiment takes *T* = 100 time stamps for training and we report the running time for each iteration in Fig 2. The running time for other two datasets are available in Fig A in S1 File. Experiments are run on Ubuntu system with Intel i7–7820X CPU @ 3.60GHz and 128G memory. Those results clearly demonstrate that inference of OSLMM is considerably faster than SLMM and SGPRN. The details of data and methods are available in Appendix D in S1 File, where we also display the same training speed comparison on two other real high-dimensional machine learning datasets. Moreover, we report the predictive performance on five real datasets in Appendix D in S1 File, which shows that OSLMM has better predictive performance on most of the datasets.

In contrast to SLMM, an assumption of OSLMM (and many other latent space methods in neuroscience, e.g., GPFA), is that the embedding subspace is fixed. There are several reasons for this assumption. The primary goal of OSLMM is to extract intrinsic latent spaces of neural population data that are clearly organized by the parametric structure of external variables commonly studied in neuroscience. This capability is critical to scientific interpretability of the latent spaces, and hence directly impacts neuroscientific conclusions. While it is true that having a fixed embedding space constrains the model, it greatly eases visualization and interpretation of resulting latent spaces, which is our desired goal. Further, the assumption of fixed embedding subspace is explored in [29], in which it is concluded that most real neural data usually lie on a low-dimensional manifold. Additionally, while SLMM is more flexible, it requires approximate Bayesian inference, which is substantially more demanding and is not guaranteed to be a better model in practice. Relatedly, SLMM typically does not scale beyond a few thousand training points and cannot deal with high dimension data, both of which are common in systems neuroscience. As such, SLMM is not applicable for most real-world data sets, and is therefore less likely to have broad adoption by the community. Finally, a model allowing time varying subspaces would easily result in overfitting issues. Indeed, we explored the assumption of fixed embedding subspace in real world datasets Table A and B in Appendix D in S1 File, where we compared data-reconstruction accuracy across five different methods for five different real world data sets. In all cases, OSLMM substantially outperformed SLMM. Indeed, OSLMM outperformed all other methods for four out of the five data sets, and was substantially better for three out of the five. The two datasets on which OSLMM was on par with GPRN (NPV) were relatively low-dimensional with trivial correlations. In summary, OSLMM balances computational efficiency with model flexibility, extends the assumption of Gaussian data to the non-Gaussian case, and, most critically, results in latent spaces that are more directly organized by the structure of exogenous variables, which is critical for interpretation and neuroscientific conclusions.

Compared with GPFA, OSLMM has two main differences. First, OSLMM introduced the log scale processes h(t) to handle the varying correlation across the time, which also extended the Gaussian observation assumption to non-Gaussian case, making the model more flexible to handle complicated relation of outputs. Second, OSLMM introduces the orthogonal constraint during the inference, which not only regularizes the model for better low-dimensional visualization, but also makes model inference more efficient.

### Data generating process for the Lorenz system

We tested the ability to recover ground-truth latent spaces in the context of the non-linear Lorenz dynamical system. Specifically, the Lorenz system describes a flow of fluids with *f*_*q*_, *q* = 1, 2, 3 as latent processes.

$$\begin{array}{c} \frac{df_{1}}{dt}=\beta_{1}\left(f_{2}-f_{1}\right),\frac{df_{2}}{dt}=f_{1}\left(\beta_{2}-f_{3}\right)-f_{2},\frac{df_{3}}{dt}=f_{1}f_{2}-\beta_{3}f_{3}\;. \end{array}$$ (6)

Lorenz sets the values *β*_1_ = 10, *β*_2_ = 28 and *β*_3_ = 8/3 to exhibit chaotic behavior as utilized in recent works [9, 30, 31].

We simulated the three-dimensional dynamics of the Lorenz system in (6) and normalized each dimensions to unit variance and zero mean. Then we generated log time-scales **H** via three different mapping functions ***h*** with different GP priors: $h_{q}\overset{iid}{∼}GP\left(0,k_{h}\right)$ with three different squared exponential covariance functions $k_{h}^{\text{short}}\left(\Delta t\right)=\text{exp}\left(-\frac{\Delta t^{2}}{2}\right)$, $k_{h}^{\text{median}}\left(\Delta t\right)=\text{exp}\left(-\frac{\Delta t^{2}}{2\;\text{exp}{\left(1\right)}^{2}}\right)$, and $k_{h}^{\text{long}}\left(\Delta t\right)=\text{exp}\left(-\frac{\Delta t^{2}}{2\text{exp}{\left(2\right)}^{2}}\right)$. Finally, we selected a random semi-orthogonal matrix **U** and generated data via ***y***_*t*_ = **U**^*T*^exp(**h**_*t*_)**f**_*t*_ + *η*_*t*_, where **h**_*t*_ = ***h***(*t*_*t*_) and the noise *η*_*t*_ are drawn from $N(0,0.1^{2}I)$.

We also developed a multiple data generating process to produce observations from the same latent trajectories (i.e., Lorenz trajectories), but with different corrupted orthogonal mappings from latent trajectories to observations. We first generated one random semi-orthogonal matrix **U** with dimension size *P* = 50 and *Q* = 3, and then generated *I* corrupted semi-orthogonal matrix **U**_*i*_, *i* = 1, …, *I*, by simulating $V_{i}=U+\sigma E_{i},E_{i}∼MN\left(0,I_{P},I_{Q}\right)$ and next extracting the closest matrix in V(P,Q) to **V**_*i*_ in the Frobenius norm, i.e. $U_{i}={\text{argmin}}_{U\in V(P,Q)}{∥V_{i}-U∥}_{F}$, where $V\left(P,Q\right)=\left\{A\in R^{P\times Q}|A^{T}A=I\right\}$ is the set of semi-orthogonal matrices. It suggests that **U** is the median subspace of {**U**_*i*_}.

### Analysis of neural data

#### Rat auditory cortex experiments

We analyzed micro-electrocorticography (μECoG) data previously collected from rat auditory cortex experiments in the Bouchard Lab [28, 32]. We analyzed the z-scored high-gamma activity of 128 simultaneously recorded μECoG channels over rat auditory cortex. High-gamma (H*γ*: 70–170Hz) activity from μECoG is a commonly-used signal containing the majority of task relevant information for understanding brain computations [33]. H*γ* primarily reflects multi-unit activity from pyramidal neurons located in infragranular layers [28]. For each experimental trial, we analyzed neural activity for a duration of 150 ms in which the auditory stimuli happened from 50 ms to 100 ms. The stimuli consisted of 240 different sounds with 8 distinct amplitudes [-70 to 0 dB attenuation] and 30 distinct frequencies from 500 Hz to 32 000 Hz [28, 32]. Each stimulus has 20 trials in the experiment. The H*γ* activity was downsampled to 400 Hz. We calculated leave-one-channel-out prediction error (accuracy), and additionally explored the latent representation of the data. We conducted both stimuli-wise and global analysis. In stimuli-wise analysis, we assume that within single stimuli the mixing coefficients **W** are shared across all trials, and different trials have their own individual latent processes. The mixing coefficients **W** are shared across all trials and stimuli in the global analysis. Moreover, we decoded the sound attenuation and sound frequency from the inferred latent functions evaluated at time with maximum norm via a linear regression.

#### Monkey arm-reaching experiments

A second dataset is obtained from monkey arm-reaching experiments and comes from [10, 34]. It consists of one full session with 2869 total trials (2295 trials for training and 574 trials for testing), 108 conditions and 182 neurons with simultaneously monitored hand kinematics. The arm-reaching task consists of a monkey reaching to presented targets while avoiding the boundaries of a virtual maze. This task has three distinct epochs: target presentation, go cue and movement onset [35]. We aligned the neural spikes from 50 ms before the move onset time to 450 after that and resampled the data at the bin size 5ms. Therefore, each trial has a multivariate spike time series with 100 time stamps. As the same in [2], we smoothed the neural activities via convolution with a Gaussian filter with 50 ms standard deviation. We conducted the global analysis where the mixing coefficients are shared across all trials and conditions. Moreover, we decoded the monkey’s hand position and velocity from the inferred latent functions with a 5-fold cross-validated ridge regression.

#### Model evaluation using leave-one-channel-prediction

The leave-one-channel-prediction is considered for model comparison. We use three-fold cross-validation of all trials and so we have three pairs of training trials and testing trials. For each pair of data, we infer the posterior samples (OSLMM) or point estimates (GPFA) of shared latent variables **U** and **h**, and model parameters Θ from training trials. Next, for each test trial, we leave one channel out of the test trial as a target and compute the posterior predictive mean of the signal of the target channel using the remaining channels with the posterior samples (OSLMM) or estimates (GPFA) of shared latent variables and model parameters from the training trials. We repeat this procedure on each test trial and each channel of the chosen test trial. We choose the sum of square error and coefficient of determination (*R*^2^) as two prediction measures for model comparison.

As single-trial neural data are regularly sampled in time, a covariance matrix generated from a stationary kernel has a Toeplitz structure. Specifically, for any Toeplitz matrix $S\in R^{T\times T}$ with constant diagonals and S_*i*,*j*_ = S_*i*+1,*j*+1_, this structure of a covariance matrix allows the corresponding GP inference in O(TlogT) and the GP prediction of variance in $O(T^{2})$ [36, 37]. Therefore, the learning complexity for our MCMC algorithm for single-trail data would be decreased to O(max(QTlogT,PT,PQ)).

#### Statistical tests

Our statistical tests were primarily based on bootstrap re-samples or cross validation of various metrics (e.g., decoding performance, differences in reconstruction accuracy, distances in latent spaces, etc.,). We used the Wilcoxon signed-rank test, and the null-hypothesis was rejected at the *α* < 0.05 level.

## Results

Inferring interpretable latent trajectories, particularly from single trial neural population recordings, is critical to understand brain computations [1]. A large class of methods assumes an autoregresive linear dynamics model in the latent process due to the computational feasibility [38, 39]. However, the assumption of linear dynamics may be overly simplistic since interesting neural computations are naturally nonlinear. Therefore, the Gaussian Process Factor Analysis method (GPFA) is a popular approach [2, 40]. Similar to GPFA, the Orthogonal Stochastic Linear Mixing Model (OSLMM) developed here imposes a general Gaussian process prior to infer latent dynamics. However, OSLMM differs from GPFA in three aspects. First, OSLMM assumes that the mixing coefficient matrix (which describes how latent functions are combined to produce observations) is time dependent, which allows modelling time-varying correlation across neurons/channels. This is critical, as it is known that the correlation structure of neural data changes over time [6, 10, 11]. Second, GPFA orthogonalisation of the mixing coefficient matrix is done as a post-processing step; in contrast, OSLMM builds the orthogonalisation of the mixing matrix into the model, arguably a more desirable modeling approach. Finally, GPFA provides only point estimates of values, while OSLMM provides samples from the posterior distribution.

We compared the OSLMM to GPFA in three settings. We first showed superior recovery performance on synthetic data generated from high-dimensional noisy observations of the Lorenz system. We next conducted analysis on two neuroscience experiments. The first analysis is on electrophysiology data from rat auditory cortex [28, 32]. The data consists of micro-electrocorticography (*μ*ECoG) high-gamma responses to tone pips of varying frequency and attenuation. The second analysis is on electrophysiology data from monkey arm-reaching [3, 34]. The data consist of simultaneously recorded single units from motor cortex while a monkey makes reaches with an instructed delay to visually presented targets while avoiding the boundaries of a virtual maze. Across these very different neuroscience contexts, we found that OSLMM extracts latent spaces that are more structured by exogenous variables (i.e., more interpretable) than GPFA, and OSLMM’s latent spaces are more predictive of exogenous variables (i.e., tone frequency, reach angle) than GPFA.

### OSLMM provides superior recovery performance on noisy observations of the Lorenz system

To examine the degree to which OSLMM can recover known latent structure from dynamic, high-dimensional, noisy observations, we first used synthetic data. We generated noisy high-dimensional observations from known dynamics produced by the Lorenz system. The simulation takes the latent dimension *Q* = 3 and the number of higher-dimensional observations *P* = 50. We compared the ability of GPFA and OSLMM to infer the dynamics of the Lorenz system from noisy, high-dimensional observations. The details of the data generating processes are provided in **Materials and Methods**. We quantitatively compared the performance of latent trajectory reconstruction by taking the difference of the root mean squared error (RMSE): ΔRMSE = RMSE_GPFA_−RMSE_OSLMM_. The larger ΔRMSE is, the better OSLMM performs compared to GPFA.

**Fig 3:**
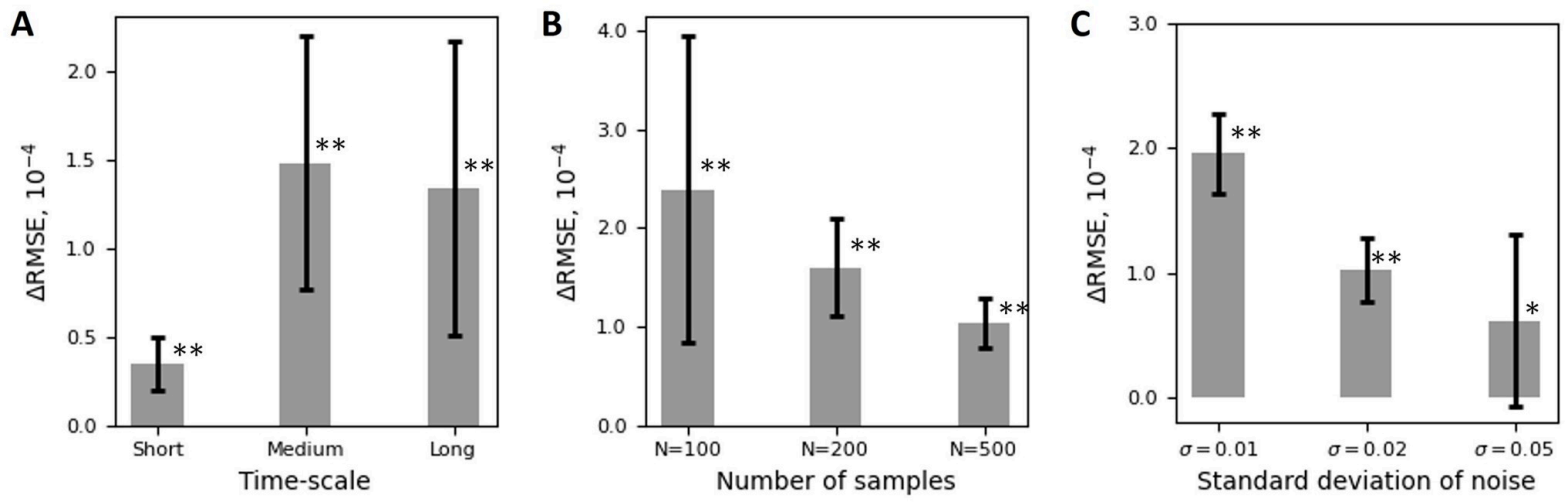
OSLMM provides superior recovery performance on noisy observations of the Lorenz system. The difference of root mean square error (ΔRMSE) of latent trajectories reconstructed from GPFA and OSLMM in three different scenarios. (A) Three data generation processes with different time scales and data size *N* = 200. (B) Three medium time-scale data generating processes in terms of various number of time steps, N = 100, 200, 500. (C) Three data generating processes in terms of different levels of noise on the subspace specified by the standard deviation of noise *σ*. Data are presented as mean and standard deviation. *: 0.01 < *p* ≤ 0.05, **: 0.001 < *p* ≤ 0.01, Wilcoxon signed-rank test.

We considered three different scenarios of the data generation process. In the first scenario, we varied the temporal length scale of the latent dynamics. We took three data generating processes with short, medium, and long timescales in terms of the length scale of Gaussian processes. We set the number of time steps *N* = 200 and considered 10 trials in each setting. In Fig 3, we plotted ΔRMSE (summarized by the mean and standard deviation) and found that OSLMM consistently had lower RMSE than GPFA (**:*p* = 1.95 × 10^−3^, Wilcoxon signed-rank test, N = 10 trials for all). In the following two scenarios, we used the medium time scale for the data generating processes. In the second scenario, we compared how GPFA and OSLMM depended on the number of data samples used for training (N = 100, 200, 500). In Fig 3B we plotted the ΔRMSE for *N* = 100, 200 and 500 samples, summarized by the mean and standard deviation over the 10 trials (**: *p* = 1.95 × 10^−3^, N = 10 trials, Wilcoxon signed-rank text). In the third scenario, we varied the amount of noise in the latent dynamics. We generated 10 trials with noise scales *σ* = 0.01, 0.02, 0.05, representing different levels of discrepancy in subspaces. We chose the number of data samples to be *N* = 200. In Fig 3C, we see that, as with previous results, OSLMM significantly outperformed GPFA (**: *p* = 1.95 × 10^−3^, *: *p* = 1.37 × 10^−2^ for *σ* = 0.01, 0.02, 0.05, Wilcoxon signed-rank text, N = 10 trials). Together, these results demonstrate that OSLMM robustly outperforms GPFA in terms of recovering underlying latent dynamics under a variety of data generating process scenarios.

### OSLMM latent spaces provide superior prediction performance for neural recordings from auditory cortex

**Fig 4:**
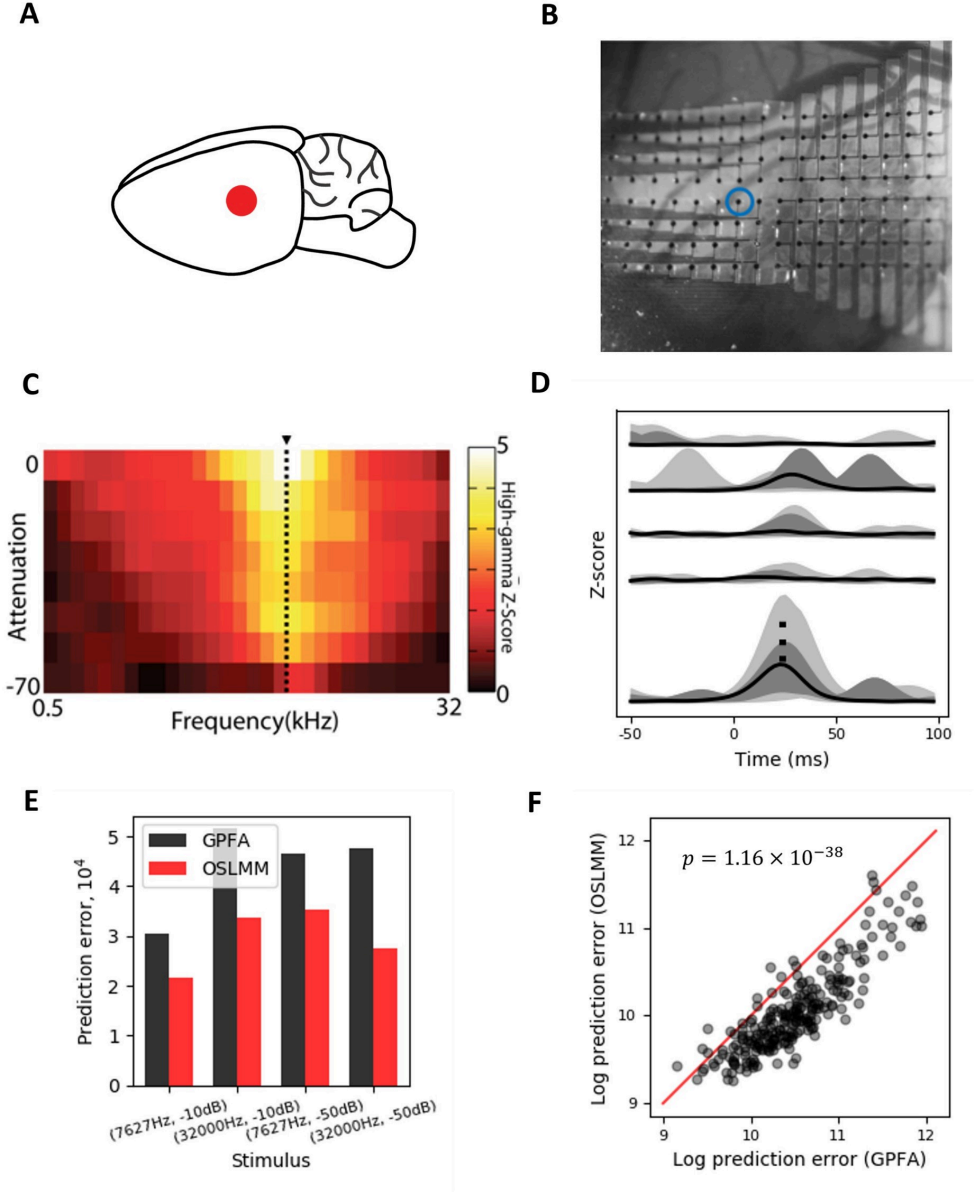
OSLMM latent spaces provide superior prediction performance for neural recordings from auditory cortex. (A) Schematic location of *μ*ECoG grid on rat brain over auditory cortex. (B) Photomicrograph of the 128-channel micro-electrocorticography array on the rat auditory cortex. The blue circle refers to one of 128 channels. (C) Heat-map of z-scored high-gamma responses from the electrode circled with blue in (B) for each frequency-attenuation pair of the presented pure-tone pip stimulus. Dashed black line demarcates the best-frequency of this electrode. (D) Functional boxplot of the z-scored activity across electrodes for a single stimuli; black lines refer to the median and light/dark grey shaded regions refer to non-outlying and central 50% regions. The stimulus takes frequency 7627 Hz and attenuation -10 dB and it starts from 0 ms and ends at 50 ms, which would affect some of electrodes in terms of Z-score displayed in this panel. (E) Prediction error on the stimuli-wise analysis for four stimuli. (F) Prediction error on the global analysis across all stimuli; each point is an electrode (p = 1.16 × 10^−38^, Wilcoxon signed-rank test, *N* = 128 channels). Panel B is reproduced from [28].

To examine the performance of OSLMM relative to GPFA on real neural data, we first analyzed data from rat auditory cortex (Fig 4A). We recorded cortical surface electrical potentials from anesthetized rat auditory cortex with customized 128-channel micro-electrocorticography (*μ*ECoG) arrays (Fig 4B) [28]. For each electrode, we extracted the high-gamma (*Hγ*: 70–170Hz) analytic amplitude from the broad-band signal, and z-scored it relative to baseline statistics (see Materials and methods). *Hγ* primarily reflects the multi-unit firing rate of infragranular pyramidal neurons [28]. The rat was presented with pure tone pips of varying frequency and amplitudes. Fig 4C displays a heat-map of the average *Hγ* neural response at the electrode demarcated in Fig 4B to each frequency-attenuation pair. We found that the *Hγ* activity at this electrode exhibited a peak-response across amplitudes to frequencies of 7627 Hz (dotted vertical line), and the magnitude of the response across frequencies increased with the amplitude of the sound. These response characteristics are similar to single-unit recordings in the auditory cortex [41]. We visually summarized the dynamics of *Hγ* activity across electrodes via the functional boxplot [15, 42]. In Fig 4D, we plotted data from the stimulus (7627Hz, -10dB)(solid black line denotes the median curve, light/dark shaded regions areas demarcate the non-outlying and central 50% regions).

We quantified the predictive performance of GPFA and OSLMM for neural activity using the prediction error in a leave-one-channel-out prediction procedure (Materials and methods). We first conducted a stimuli-wise analysis, in which mixing coefficients are shared across all trials of a single stimulus, and different trials have their own latent process. For this analysis, we chose four stimuli as the combinations of two attenuations −10*dB* and −50*dB* and two frequencies 7627 Hz and 32 000 Hz. We considered the latent dimension *Q* = 5 and independently ran GPFA and OSLMM on the four subsets of the data. The prediction errors for the four stimuli are reported in Fig 4E. For all four stimuli, the prediction error of OSLMM is consistently smaller than that of GPFA. Moreover, we analysed the relation between predictive performance and latent dimension size *Q* in Appendix E in S1 File with prediction performance shown in Fig C in S1 File. This shows that for most combinations of the stimuli and latent dimension size, the prediction error of OSLMM is smaller than that of GPFA, and *R*^2^ of OSLMM is larger than that of GPFA. We next compared GPFA and OSLMM in a global analysis across all 4800 trials and utilized a leave-one-channel-out prediction. In contrast to the stimuli-wise analysis above, here the mixing coefficients are shared across all trials and stimuli. For each electrode, we reported the prediction error (sum of squared error) on the logarithmic scale in Fig 4F. We found that the prediction errors for the overwhelmingly vast majority of electrodes from OSLMM were smaller than those from GPFA (Wilcoxon signed-rank test on the log prediction error, p = 1.16 × 10^−38^, *N* = 128 channels). Moreover, we decoded (with ridge-regression) both sound attenuation and sound frequency using the latent functions from OSLMM, GPFA, and PCA (Materials and methods). We conducted bootstrap sampling with 100 bootstrap replicates and found that for decoding of attenuation, the mean and 95% confidence interval of R^2^ scores were 0.45(0.42, 0.51), 0.64(0.60, 0.69) and 0.84(0.80, 0.86) for PCA, GPFA and OSLMM, while for decoding of frequency, R^2^ scores were 0.03(0.03, 0.04), 0.06(0.04, 0.09) and 0.50(0.45, 0.52) for PCA, GPFA and OSLMM. This illustrates that latent functions of OSLMM can be better linear decoders (at the time of max response) for those two exogenous stimulus parameters. Together, these results indicate that OSLMM extracts latent functions that are better predictors of neural data and decoders of exogenous variables.

### OSLMM latent spaces extracted from auditory cortex population activity are structured by external stimuli

Having demonstrated that OSLMM achieves superior prediction performance of neural activity compared to GPFA, we next examined the structure of the inferred latent spaces. Decades of studies utilizing single-unit recordings, LFP, ECoG, fMRI, etc., across diverse mammalian species (mice, rats, monkeys, humans) have demonstrated that primary auditory cortex neural responses are monotonically modulated by the amplitude of the stimuli, and are tuned to a preferred sound frequency [43]. Therefore, we hypothesized that inferred latent dynamics would be structured by these properties of the stimulus.

To test this hypothesis and to compare the ability of OSLMM and GPFA to extract latent spaces with the hypothesized structure, we applied both GPFA and OSLMM to jointly model the trials of all different stimuli, and explored the structure of the latent spaces. For both methods, we set the latent dimension *Q* = 5, and then inferred the latent functions of all trials. Latent functions are rotated to maximize the power captured by each latent in decreasing order. Finally, we averaged the orthonormalized latent functions by either sound attenuation or sound frequency over trials to ease visualizations.

**Fig 5:**
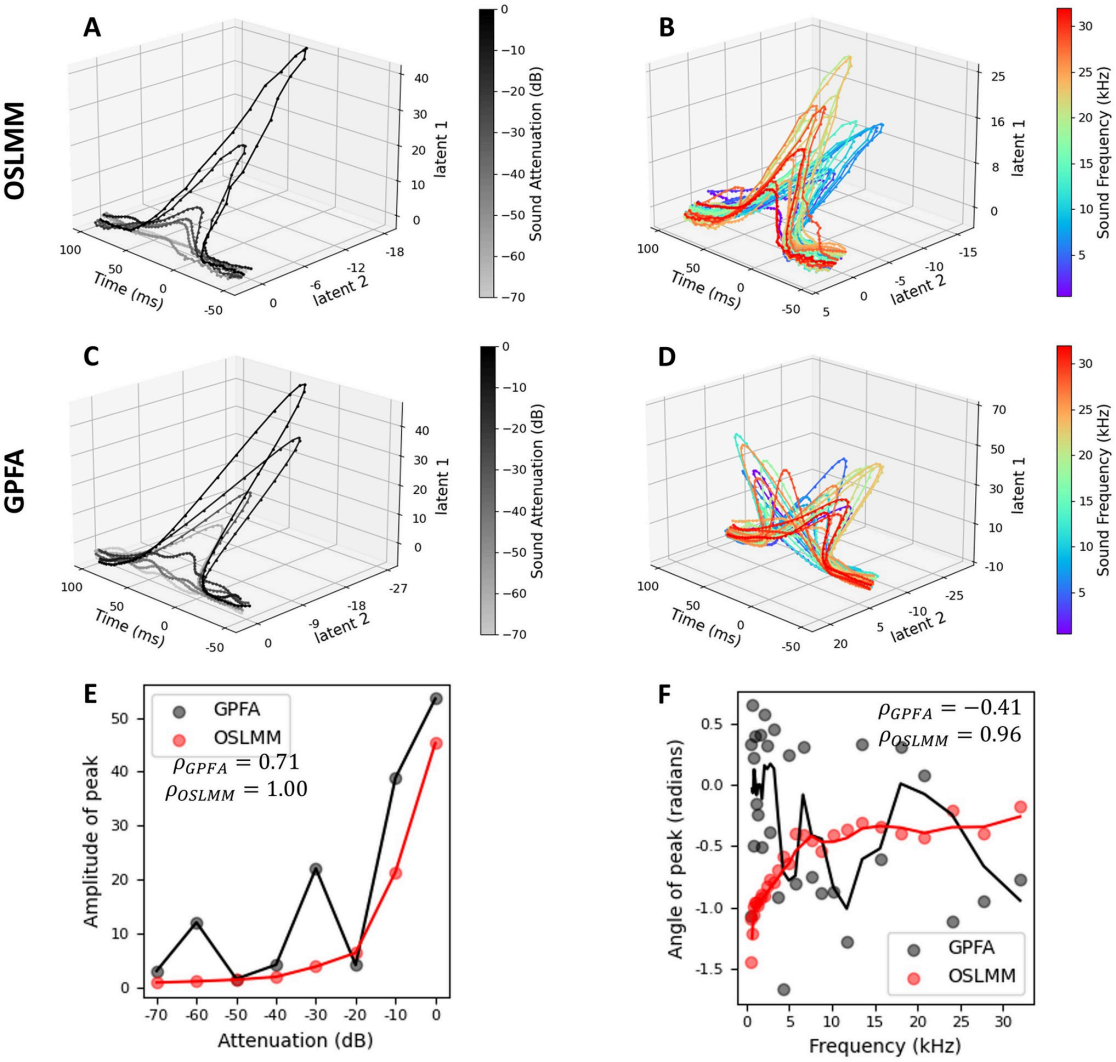
OSLMM latent spaces extracted from auditory cortex population activity are structured by external stimuli. (A, C) Trial-averaged latent neural trajectories for all attenuations with a fixed frequency 7627 Hz for OSLMM and GFPA respectively; (B, D) Trial-averaged latent neural trajectories for all frequencies with a fixed attenuation (−10dB) for OSLMM and GPFA respectively. (E) Stimulus attenuation vs. the amplitude of the latent trajectory. (F) Stimulus frequency vs. the angle of latent trajectory. Lines in (E,F) are a moving average.

We plotted the averaged orthonormalized latent functions for all eight stimuli attenuations for a fixed frequency of 7626 Hz in Fig 5A for OSLMM and Fig 5B for GPFA. Likewise, we plotted the averaged orthonormalized latent functions for all thirty frequencies with a fixed attenuation of −10 dB in Fig 5C for OSLMM and Fig 5D for GPFA. We found that OSLMM latent spaces had a monotonic ordering of both the different sound attenuations (Fig 5A) and sound frequencies (Fig 5C). In contrast, GPFA latent spaces did not have this property (Fig 5B and 5D). Specifically, the OSLMM latent trajectories for a single frequency and different sound attenuations extended in one direction (Fig 5A) with a magnitude that increased monotonically with increasing sound amplitude(grey-to-black), while the GPFA latent trajectories had mixed ordering (Fig 5B). Likewise, different sound frequencies (blue-to-red with increasing frequency) at a single attenuation smoothly transitioned across angles of the OLSMM latent trajectories (Fig 5C), but were highly intermixed in the GPFA latent trajectories (Fig 5D).

We quantified these effects by extracting the amplitude of the latent trajectories as a function of sound amplitude, and the angle of the latent trajectories as a function of sound frequency. We plotted the sound attenuation vs. the amplitude of latent trajectory in Fig 5E and plotted the sound frequency vs. the angle of latent trajectory in Fig 5F. These plots show OSLMM latent trajectories have a perfectly monotonic relationship between sound attenuation and trajectory amplitude (Fig 5E, red), and a nearly perfect monotonic relationship between sound frequency and trajectory angle (Fig 5F, red). However, the relationship between GPFA trajectories and these stimulus properties is less pronounced (Fig 5E and 5F black). We quantified these effects with the Spearman rank correlation (*ρ*) for GPFA and OSLMM between sound attenuation and trajectory amplitude, and between sound frequency and trajectory angle (amplitude: *ρ*_*OSLMM*_ = 1, *ρ*_*GPFA*_ = 0.71; frequency: *ρ*_*OSLMM*_ = 0.96, *ρ*_*GPFA*_ = −0.41, Fig 5E and 5F). To show the statistical significance of Spearman rank correlation, we conducted bootstrap sampling with 100 bootstrap replicates, and then conducted a Wilcoxon signed-rank test using the bootstrap *ρ*. We found that the difference of *ρ* values between GPFA and OSLMM is statistically significant (*p* = 8.11 × 10^−17^ for sound attenuation, *p* = 3.90 × 10^−18^ for sound frequency). We displayed the latent trajectories with latent dimension *Q* = 10 in Fig D in S1 File and those with *Q* = 15 in Fig E in S1 File (Appendix F in S1 File). These results illustrate the stronger monotonic relationship OSLMM latent spaces than in GPFA latent spaces, and are robust to the choice of latent dimensions size. We further visualized the latent neural trajectories of the auditory responses with and without the time varying scale factor (W(t)) (Fig F in S1 File). We found that the differences were entirely in the magnitude of projection, and the geometry of the trajectories with respect to each other and their relationship to the stimulus parameters (attenuation, frequency), were essentially unaltered. Together, these results demonstrate that the OSLMM latent trajectories reflect distributed auditory cortical population response properties that are not captured by GPFA.

### OSLMM’s latent spaces extracted from motor cortex are predictive of behavior

**Fig 6:**
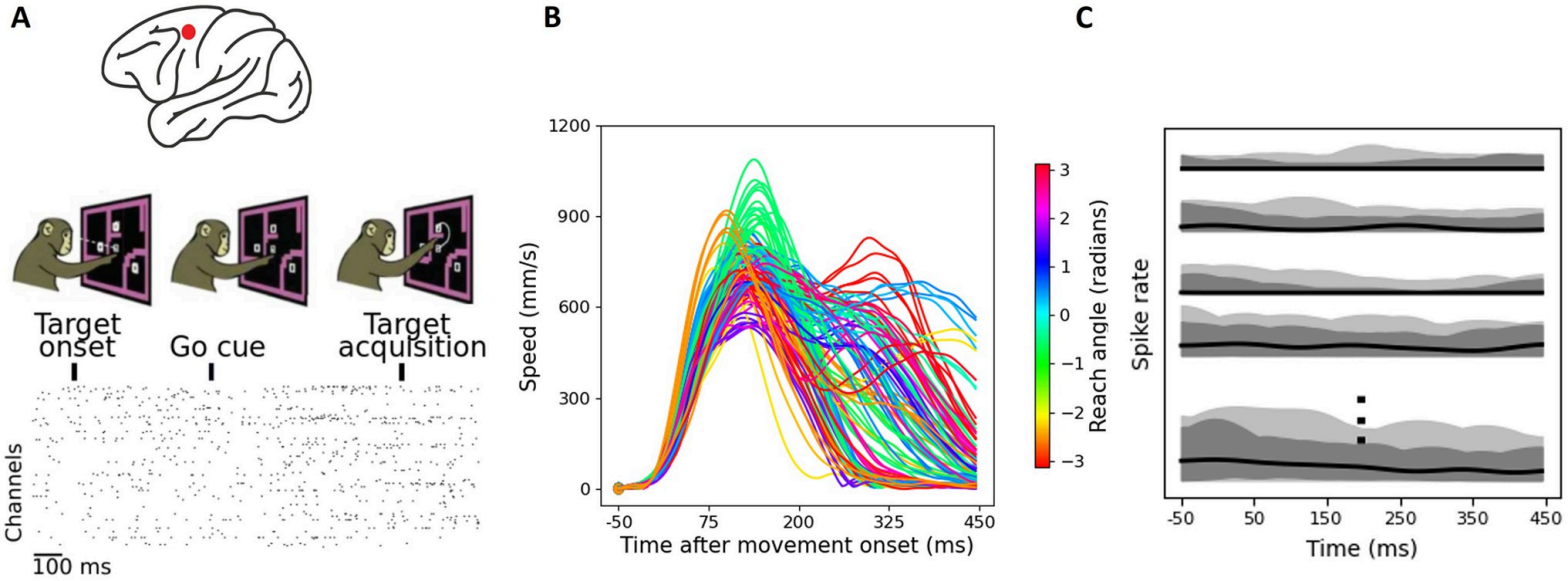
OSLMM’s latent spaces extracted from motor cortex are predictive of behavior. (A) (top) Schematic location of motor cortex recordings on non-human primate brain; (middle) schematic of maze reaching task; (bottom) raster plot of extracted neural spike data from monkeys’ motor cortex (each tick mark corresponds to detected spike time). (B) The average speed profiles for 108 conditions. Each profile is colored according to the average reach angle. (C)Functional boxplots of the smoothed spike rates for each condition where black lines refer to the median curve and light/dark grey shaded areas refer to non-outlying region and 50% central region. It displays the general the spike rates pattern across all conditions. Panel A is reproduced from [10, 34].

Recordings from monkey motor cortex during reaching tasks have emerged as an important test-bed for latent space methods [2, 8, 44]. Therefore, we next compared OSLMM to GPFA on multiple single-unit recordings from motor cortex (182 neurons) while the monkey performed a delayed reaching task with obstructing barriers forming a maze (2869 trials total) [10, 34] (Fig 6A). Data and analysis details are available in Materials and methods. The average hand speed profiles for 108 conditions colored by average reach angle are displayed in Fig 6B. We visualized the smoothed the spike rates for several conditions across trials in Fig 6C using functional boxplots (black lines: median; light/dark shaded area: non-outlying region and central 50% region).

We first compared the ability of OSLMM to GPFA and PCA to extract latent structure from the population neural activity that was predictive of the monkeys reaching behavior. OSLMM, GPFA, and PCA were applied to the population spike rates (182 neurons) across all 2869 trials of reaches across all reach angles/speeds. The orthogonalized latent functions were estimated, and then rotated to maximize the power captured by each latent in decreasing order. We decoded the monkey’s hand position and hand velocity solely from the inferred latent functions from OSLMM, GPFA, and or PCA with cross-validated ridge regression (we set the latent dimension size *Q* = 6 for all methods). To estimate the uncertainty of R^2^ scores, we conducted bootstrap sampling with 100 bootstrap replicates. For hand position, the mean and 95% confidence interval of decoding R^2^ scores for PCA, GPFA and OSLMM are 0.535 (0.527, 0.543), 0.574 (0.567, 0.582) and 0.602 (0.594, 0.610). In terms of hand velocity, those metrics are 0.504 (0.497, 0.511), 0.518 (0.512, 0.525), and 0.544 (0.538, 0.550) for PCA, GPFA and OSLMM respectively. We further quantified the dynamics of structure in the latent spaces by measuring the distances between individual condition trajectories (Appendix G in S1 File) with distance plot in Fig H in S1 File. We found that both maximum mean and standard deviation of trajectory distances were significantly larger for OSLMM than GPFA (p = 3.90 × 10^−18^ and p = 4.67 × 10^−18^, Wilcoxon signed-rank tests, *N* = 100 resamples for each). These results indicate that OSLMM trajectories for different reaches had more distance between them than GPFA trajectories. Furthermore, the dynamics of the distances from OSLMM trajectories for different reach angles were grouped together, and then diverged to a maximum spread during the middle of the reach (during which reach velocity is most heterogenous, Fig 6B), and then converged again. GPFA trajectory distances did not exhibit such behaviorally relevant dynamics. Together, these results demonstrate that OSLMM provides significantly better predictive performance of reaching kinematics than GPFA or PCA.

### OSLMM latent spaces extracted from motor cortex population activity are structured by reach kinematics

**Fig 7:**
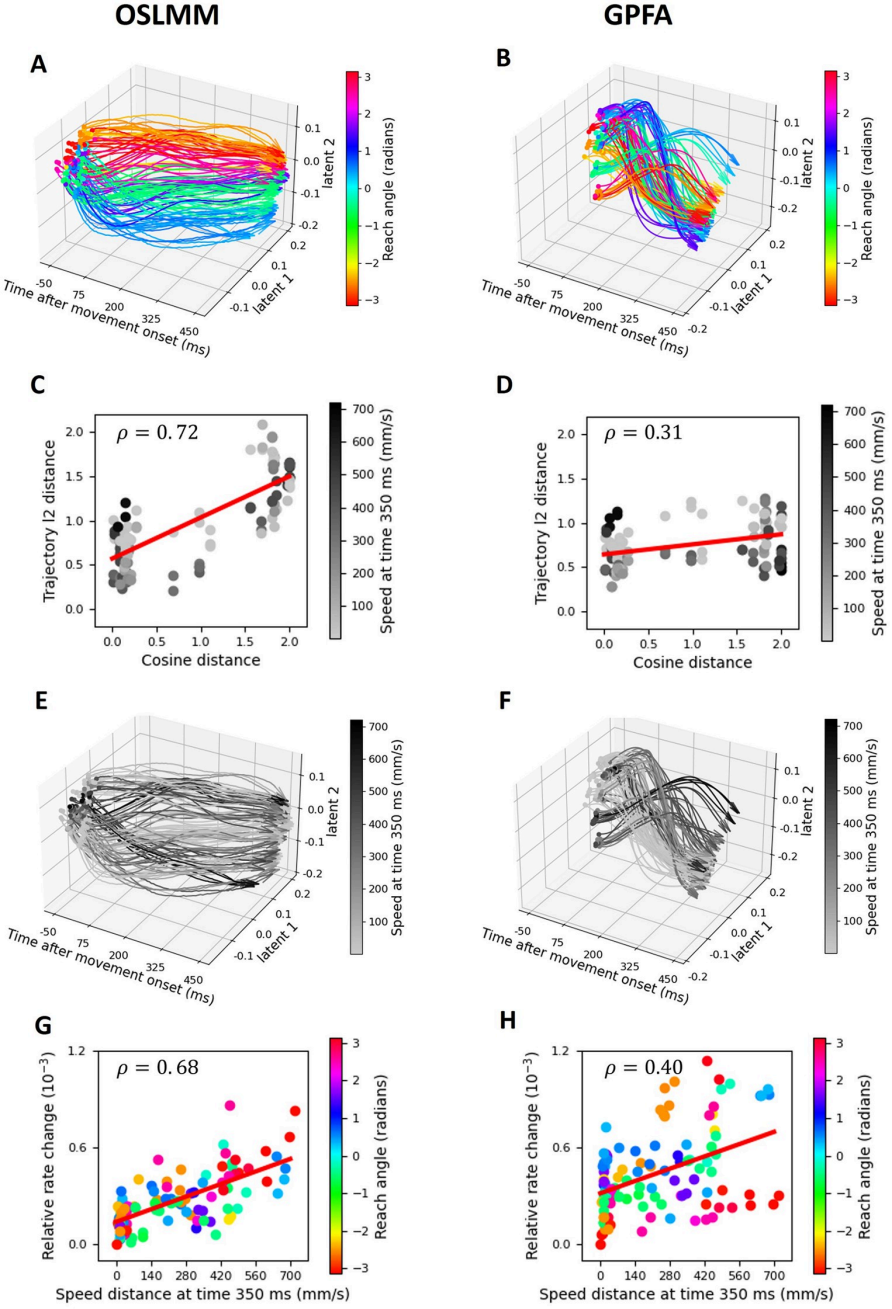
OSLMM latent spaces extracted from motor cortex population activity are structured by kinematics. (A,B) Trial-averaged latent trajectories for all reach conditions colored by reach angle for OSLMM (A) and GPFA (B). (C,D) Scatter plot of cosine distance between reach angles vs. the distance between latent trajectories for OSLMM (C) and GPFA (D). Each point is colored (gray-scale) according to the speed (at 350ms) of the reach. (E,F) Trial-averaged latent trajectories but for all reach conditions colored (grey scale) by the speed at 350ms for OSLMM (E) and GPFA (F). (G,H) Scatter plot of difference in speeds vs. the rate of change of latent trajectories for OSLMM (G) and GPFA (H). Each point is colored according to the average angle of the reach.

Having demonstrated the superior predictive performance of OSLMM vs. GPFA for reach kinematics, we next examined if and how neural trajectories extracted from motor cortex were structured by reach kinematics. We first examined if and how latent trajectories were structured by the angle of the reach. We plotted the first two latent components vs. time and colored the latent trajectories by reach angle for OSLMM (Fig 7A) and GPFA (Fig 7B). We observed that reach angle clearly structured the latent trajectories in OSLMM. In particular, for the second latent dimension, as the value varies from low to high, the color varies from blue to red via green or purple. In other words, as the value of the second latent dimension increases, the reach angles are deviating from zero radians. In contrast, for GPFA, the colors are mixed throughout time, and it is hard to see any relation between latent trajectories and reach angles (colors). To quantify this relationship, we calculated the *ℓ*_2_ distance between individual latent trajectories and the baseline trajectory (defined as the average of all trajectories with reach angle within [-0.5, 0.5] radians). We plotted the distance between trajectories as a function of the angular distance (cosine distance) of the corresponding reaches for OSLMM (Fig 7C) and GPFA (Fig 7D). Here, we indicated the speed of the reaches as the grey-scale of the points. We found that, for OSLMM (Fig 7C), as the distance between reach angles increased, so to did the distance in latent space (Spearmean *ρ*_*OSLMM*_ = 0.72 with p-value 2.54 × 10^−18^). This relationship was much weaker for GPFA (Fig 7D, Spearman *ρ*_*GFPA*_ = 0.31 with p-value 1.12 × 10^−3^). There was no visually salient modulation by reach speed for either GPFA or OSLMM for this metric of latent trajectories. The difference in Spearman *ρ* values was statistically significant (*p* = 3.90 × 10^−18^, Wilcoxon signed-rank test, *N* = 100 resamples).

We next examined if and how reach speed impacted latent trajectories. We first colored the latent trajectories by the speed of the reach at 350ms (where speed profiles had high variance, Fig 6B) into grey-scale for OSLMM (Fig 7E) and GPFA (Fig 7F). For OSLMM, the latent trajectories corresponding to slower speeds (light grey) had less movement over time than did the trajectories corresponding to higher speeds (black)(Fig 7E). That is, there were more rapid dynamics of latent trajectories for more rapid reaches. In contrast, for GPFA, there appeared to be no systematic effect on the latent dynamics for different reach speed (Fig 7F). To quantify this relationship, we first defined the change in latent space as the difference between the position at time 200 ms to position at time 300 ms. The relative rate of change is then the difference in the rate of change for each condition and the baseline condition, where the baseline condition is defined as the reach with the minimum speed at time 350 ms. We plotted the rate of change against the difference between the speed for each condition and the baseline condition at time 350ms for OSLMM in Fig 7G and for GPFA in Fig 7H. We found that OSLMM had a more robust relationship between speed and latent trajectories (Spearman *ρ*_*OSLMM*_ = 0.68 with p-value 6.42 × 10^−16^) than did GPFA (Spearman *ρ*_*GPFA*_ = 0.40 with p-value 1.63 × 10^−5^). There was no visually salient modulation by reach angle for either GPFA or OSLMM for this metric of latent trajectories (coloring of points). The difference between Spearman *ρ* values was statistically significant (*p* = 3.90 × 10^−18^, Wilcoxon signed-rank test, *N* = 100 bootstrap resamples). We further visualized the latent neural trajectories of the motor cortex with and without the time varying scale factor (W(t)) (Fig G in S1 File). In contrast to the auditory cortex trajectories, the geometry of latent neural trajectories were substantially different between the two. In particular, the unscaled trajectories were much more tangled and had less organization with respect to the reach angle compared to the scaled trajectories. Together, these results demonstrate that latent spaces of motor cortex neural populations during reaches are structured by the kinematics of the reach.

**Fig 8:**
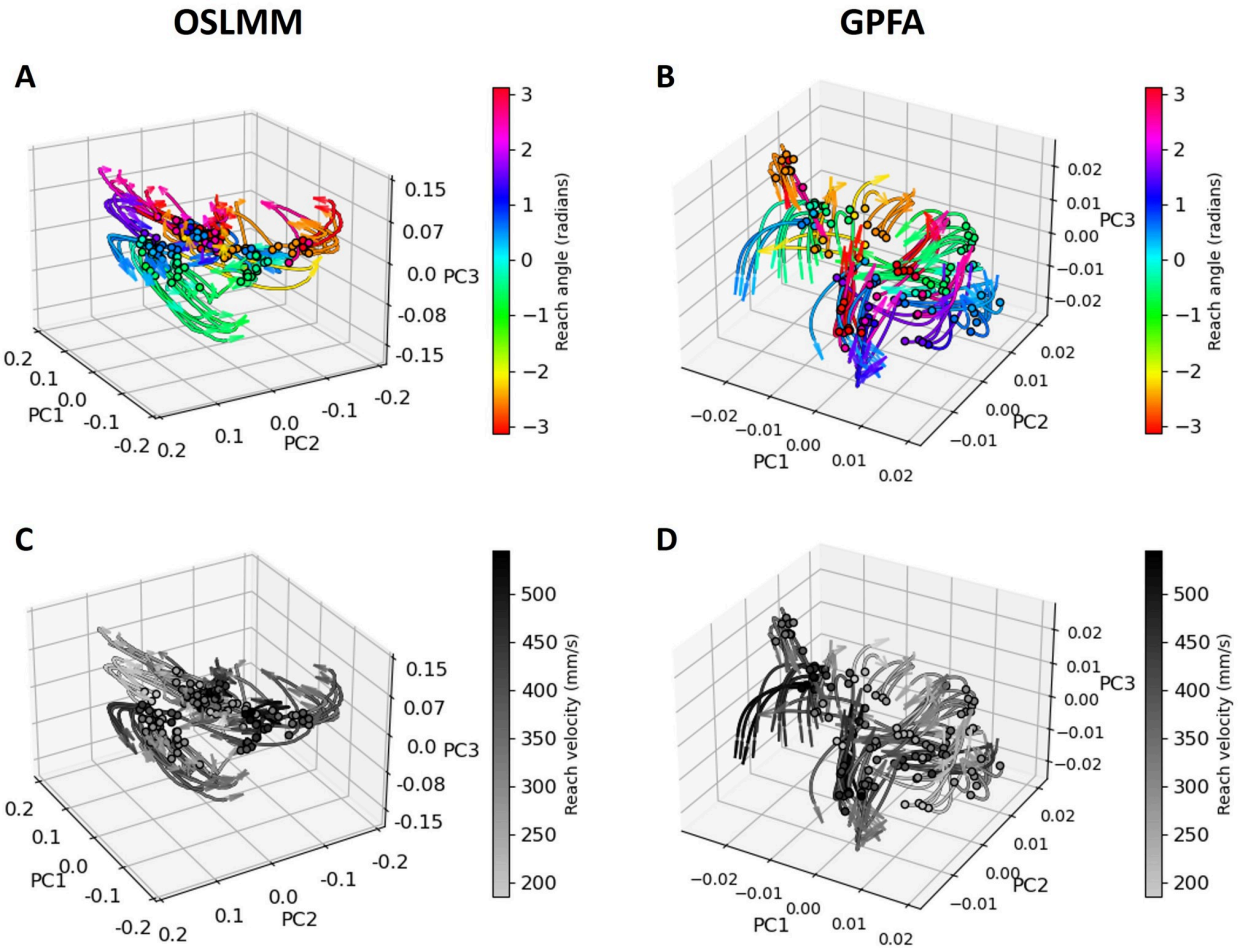
OSLMM latent spaces of motor cortex dynamics are structured by reach angle. (A, C) First three jPCA dimensions from the OSLMM extracted latent space; (B, D)First three jPCA dimensions from the GPFA extracted latent space. We encoded reach angle as color in (A,B) and reach speed into grey scale in (C, D).

Finally, to provide further insight into the structure of latent dynamics in motor cortex, we conducted jPCA [3] based on the extracted latent trajectories from GPFA and OSLMM. jPCA finds latent directions of maximal rotational dynamics, and we visualized neural trajectories in the first three jPCs for the first 150ms in Fig 8. As above, we encoded reach angles into colors (Fig 8A and 8B) and encoded reach speeds into grey scale (Fig 8C and 8D). Visually, we observed that the reach angles imparted structure to the jPCA trajectories extracted from OSLMM (Fig 8A), but not GPFA (Fig 8B). More specifically, through the counter-clockwise direction, the color (i.e., angle) in Fig 8A varied from green to yellow, orange to red, and purple to blue, which matches the dehttps://dandiarchive.org/dandiset/000128creasing order (looped) in reach angles. However, no clear dependence between jPCA trajectories and reach angles was observed in Fig 8B. The jPCA trajectories were not visually structured by speed for either OSLMM or GPFA (Fig 8C and 8D). Together, these results indicate that latent rotational dynamics of motor cortex neural populations during reaches can be structured by the kinematics of the reach.

## Discussion

We developed a new Bayesian multi-output regression framework, the orthogonal stochastic linear mixing model (OSLMM). OSLMM can capture input-dependent correlations across outputs and enables accurate prediction by utilizing an adaptive mixing mechanism, where mixing coefficients depend on inputs. Moreover, by imposing orthogonality constraints on the coefficient matrices, MCMC inference scales linearly with the output dimension and the number of latent functions. Together, these innovations enable application to large datasets with complicated input-dependent correlations across many outputs. We demonstrated the numerical superiority of OSLMM in various real-world benchmark datasets. Finally, we used OSLMM for analysis of diverse single-trial neural data, demonstrating that it provides not only better prediction performance but also extracts latent spaces structured by exogenous variables, enhancing neuroscientific interpretation.

Powered by new technologies, neuroscientists are recording from ever larger populations of neurons across broad spatio-temporal scales. Such datasets simultaneously bring with them the promise of improved insight into brain mechanisms producing complex functions, and the challenge of developing new analytic techniques to provide that insight. However, if and how neuroscientific conclusions about the structure (or lack-there-of) of latent neural population activity depends on the methods used is rarely considered. We applied OSLMM to extract latent spaces in two very different neurosicence datasets: *μ*ECoG recordings from rat auditory cortex in response to pure tone pips (collected in our lab), and multiple single-unit recordings from monkey motor cortex during arm reaches from [10, 34]. In both datasets, we find that OSLMM latent spaces provided a robust basis for diverse prediction tasks. More importantly, we found that OSLMM extracted latent spaces were directly structured by the exogenous variables, which in some cases changed neuroscientific interpretation relative to GPFA.

*μ*ECoG recordings from rat auditory cortex in response to pure tone pips provide a powerful test-bed for comparing latent spaces extracted by different methods. The response properties of single *μ*ECoG channels are well matched to the response properties of single-neurons in the cortical column underneath the electrode: responses are tuned to a single best frequency, and responses increase with increasing sound amplitude [28, 41, 43]. In contrast to conventional electrophysiological recording techniques, the *μ*ECoG recordings utilized here spanned the entire extent of rat primary auditory cortex, as well as secondary and tertiary auditory fields [28]. Across the auditory cortex, the representation of sound frequency is spatially organized (’tonotopy’), which is a recurring functional organizational principle in mammals [43]. Across electrodes, sound amplitude and sound frequency are primary sources of response variance, providing strong expectation that latent trajectories extracted from the population activity should be organized by those stimulus features. Indeed, we found that latent population activity was strongly organized by sound frequency and amplitude. However, this finding depended on the method used to extract latent spaces, as GPFA latent spaces were not robustly organized by both of these features. Thus, the absence of structured latent spaces is, in and of itself, not sufficient to draw neuroscientific conclusions. This should come as no surprise, as it is well known that negative scientific results (e.g., lack of structure) can emerge from many sources (e.g., choice of methods) and may not reflect scientific reality.

Recent perspectives on motor cortex argue that, in contrast to a representational perspective, it is best thought of as dynamical system that drives behavior, and should be viewed through the lens of latent spaces extracted from neural population activity activity [1, 3, 45]. This perspective is supported by findings of strong rotational dynamics in the latent neural population activity, despite the fact that the behavior itself (arm reaches) are not cyclic [3]. It is further argued that the kinematic parameters of produced reaches impart little-to-no structure to rotational latent population dynamics [3]. However, as we showed for recordings from auditory cortex, lack of organization of latent trajectories by exogenous variables can simply be a consequence of the method used to extract the latent spaces from the data, not necessarily a statement of the latent structure in the data *per se*. Indeed, many studies have found that the activity of single neurons in motor cortex can be decently explained by specific kinematic parameters of the behavior, such as reach angle and speed [46–48]. Thus, we hypothesized that the latent structure of motor cortex population activity during reaching is shaped by reach kinematics. We found that latent spaces were structured by both reach angle and speed, and moreover, rotational dynamics (extracted via jPCA) are structured by reach angle. As with the auditory cortex results, these findings were robustly revealed by OSLMM and but not GPFA.

While we emphatically agree with a dynamical systems perspective of brain activity, we believe that the representational and dynamical perspectives are not as incompatible as has been previously claimed. That is, the dynamics of population activity can be structured by the features of exogenous variables (e.g., sound frequency and reach angle) represented by individual neurons. Indeed, we have demonstrated that latent population trajectories can simultaneously be structured by the properties of individual neurons (i.e., reflect reach angle, which is known to modulate single-neuron responses) and reveal properties not obvious at the single-neuron level (e.g., rotations). These results emphasize the potential perils of interpreting negative results from latent spaces and advocate for application of the same scientific rigor used to evaluate negative experimental results. More broadly, we argue that connecting latent spaces/dynamics of population neural activity to well characterized properties of single-neurons provides important neurobiological insight. This insight may ultimately permit understanding how specific neurons contribute to the generation of population dynamics based on their transcriptomic, physiologic, and connectomic properties. Such understanding is critical for targeted interventions to alleviate neurological disorders.

Interestingly, in both auditory cortex and motor cortex, we observed that different parameters of the exogenous variables were reflected by distinct characteristics of the latent trajectories. Specifically, in the auditory cortex, sound frequency modulated the angle of latent trajectories while sound amplitude modulated the magnitude of those trajectories. Likewise, in the motor cortex, reach angle modulated the location of latent trajectories and rotational dynamics, while reach speed modulated the rate of change of latent trajectories (but not rotational dynamics). Having independent exogenous features impart different structure in latent spaces may provide a basis for separate information channels that are read-out by downstream/upstream neurons. Thus, such latent spaces may be utilized for information transmission across long-range anatomical connections that form an information bottleneck [49, 50]. Future work relating sparse codes to such latent structure could shed light on this conjecture [50, 51].

A variety of methods for extracting latent structure from neural population data have been recently introduced. LFADS (Latent Factor Analysis via Dynamical Systems) [8] is a sequential model based on a variational auto-encoder, which leverages the RNN to model the temporal dependence in the latent space. However, the latent space learned by LFADS was not used to visualize latent neural trajectories, but more to ‘process’ the data for subsequent input to linear subspace methods (PCA and jPCA) [3]. Similar to LFADS, NDT refers to Neural Data Transformers, a non-recurrent neural network similar to LFADS that replaces the RNN encoder decoder with Transformer instead [52]. MINT refers to “mesh of idealized neural trajectories” [53]. It simply uses a state-transition lookup table to model the latent dynamics and proposes two conditional functions to jointly model both spiking activity and the behavioral statistics. In contrast to LFADS and NDT, OSLMM is a non-neural network model that focuses on extracting intrinsic latent trajectories that are organized by the parametric structure of exogenous variables, instead of model predictive performance. MINT focuses on latent representations learned from both behavior trajectory and spiking observations, while OSLMM targets representations for only spiking activities. That is, OSLMM identifies intrinsic latent representations, while MINT was directly designed to improve the performance of brain computer interfaces by modeling both the extrinsic behavior and neural data simultaneously.

Compared with other Gaussian process based methods, some advantages and differences of the OSLMM are addressed. First, similar to most linear models of coregionalizations [15, 54], GPFA assumes fixed correlations between neurons. However, in neural data sets, it has been observed that there are time-dependent changes in correlation structure that are temporally aligned to the stimulus, violating this assumption [6, 10, 11]. OSLMM addresses this issue by employing an adaptive linear function of latent functions to admit correlation structure changes over time. Recent works [15, 17] have also illustrated that the adaptive linear projection structure can deal with input-dependent correlation, scale, and smoothness of outputs. Second, similar to the Orthogonal Instantaneous Linear Mixing Model (OILMM) in [20], OSLMM assumes the coefficient matrices in the coupling between latent dynamics and observations are orthogonal, but OSLMM enables the coefficient matrices to vary across inputs while the OILMM does not. Moreover, instead of imposing orthogonality on coefficients *post-hoc*, as in the GPFA, OSLMM directly imposes orthogonality on stochastic coefficients. The orthoganility constraint enables scaling inference by breaking down the high dimensional prediction problem into independent single-output problems and using efficient MCMC to sample the orthogonal space on the Steifel manifold. Both the conditional linear and orthogonality properties of OSLMM contribute to enhanced extraction of interpretable latent spaces. Lastly, we note that, like the Gaussian process regression network [21], OSLMM is strictly a non-Gaussian model due to its adaptive mixing mechanism. Future work could derive variational inference for OSLMM to overcome the expensive sampling of all latent functions when the number of samples is very large.

In summary, we have demonstrated that OSLMM enables Bayesian inference in time-series datasets with large-numbers of latent dimensions, provides superior performance on diverse prediction tasks, and reveals organization of unsupervised latent spaces by exogenous variables. As with neuroscience, many other fields of biology are benefiting from the collection of data from large numbers of sensors over time [55]. Therefore, these results indicate that OSLMM will be beneficial for analysis of many high-dimensional biological time-series datasets where data-driven discovery of complex latent structure is crucial for understanding the data generation process.

## Supporting information

S1 File Appendix A. Hyper-parameter learning for SLMM. Appendix B. Theoretical proofs for sufficient statistics. Appendix C. Hyper-parameter learning for OSLMM. Appendix D. Prediction comparison on real datasets. Table A. Predictive mean absolution error of five methods on three real datasets, **Equilty**, **PM2.5** and **Neural**. The results were summarized by mean and standard deviation over 5 runs. Fig A. Training speed of SLMM, OSLMM and SGPRN inference algorithms on **Equity** data (A) and **PM2.5** data (B). We show the running time per iteration in the setting with different number of latent functions. Table B. Predictive mean absolution error of five methods on three real datasets, **Jura** and **Concrete**. The results were summarized by mean and standard deviation over 5 runs. Fig B. First two principle angles derived from the SLMM model for five real data. First principal angle is on left while the second principal angle is on right. Appendix E. Analysis between predictive performance and latent dimension size in ECoG dataset. Appendix F. Analysis between latent representation performance and latent dimension size in ECoG dataset. Fig C. Prediction performance on leave-one-channel-prediction task on different latent dimension size *Q* = 2, 4, 8 and 16. S1, S2, S3 and S4 represent four stimuli with paired of conditions (7627Hz, -10dB), (32000Hz, -10dB), (7627Hz, -50dB) and (32000Hz, -50dB). Fig D. Inferred orthonormalized latent functions from OSLMM and GPFA for all stimuli with *Q* = 10. (A-B) Eight stimuli for all attenuation with a fixed frequency 7627 Hz averaged by trials. (A) OSLMM; (B) GPFA); (C-D): The same type of inferred orthonormalized latent functions for OSLMM (C) and GPFA (D) but for all frequencies with a fixed attenuation -10 dB averaged by trials. Moreover, we conducted linear regression between the peak of latent functions and exogenous variable (attenuation or frequency). The *R*^2^ scores for OSLMM/GPFA are 0.71/0.61(Frequency: 7627) and 0.28/0.06(Attenuation: -10). Appendix G. Latent trajectories with/without scaling. Fig E. Inferred orthonormalized latent functions from OSLMM and GPFA for all stimuli with *Q* = 15. (A-B)Eight stimuli for all attenuation with a fixed frequency 7627 Hz averaged by trials. (A) OSLMM; (B) GPFA); (C-D): The same type of inferred orthonormalized latent functions for OSLMM (C) and GPFA (D) but for all frequencies with a fixed attenuation -10 dB averaged by trials. Moreover, we conducted linear regression between the peak of latent functions and exogenous variable (attenuation or frequency). The *R*^2^ scores for OSLMM/GPFA are 0.85/0.62(Frequency: 7627) and 0.50/0.06(Attenuation: -10). Fig F. Latent trajectories of ECoG auditory responses with (A and C) and without (B and D) the time varying scale factor. The log scale trajectories of ECoG auditory responses ranked by the corresponding variance (E). Fig G. Time varying scale analysis in motor cortex data. Latent trajectories with (A) and without (B) time-varying scale. The log scale trajectories of motor cortex responses ranked by the corresponding variance (C). Distance plots for latent trajectories. Fig H. Distance plots of latent trajectories for OSLMM (A) and GPFA (B). The mean and one standard deviation below and above it for the point-wise distances are provided.
(PDF)
