# Supplementary material for: Bayesian inference of structured latent spaces from neural population activity with the orthogonal stochastic linear mixing model
Source: PLoS Comput Biol. 2024 Apr 26;20(4):e1011975. doi: 10.1371/journal.pcbi.1011975 (PMC11078355; doi:10.1371/journal.pcbi.1011975)
Supplement: S1 File — Appendix A. Hyper-parameter learning for SLMM. Appendix B. Theoretical proofs for sufficient statistics. Appendix C. Hyper-parameter learning for OSLMM. Appendix D. Prediction comparison on real datasets. Table A. Predictive mean absolution error of five methods on three real datasets, Equilty, PM2.5 and Neural. The results were summarized by mean and standard deviation over 5 runs. Fig A. Training speed of SLMM, OSLMM and SGPRN inference algorithms on Equity data (A) and PM2.5 data (B). We show the running time per iteration in the setting with different number of latent functions. Table B. Predictive mean absolution error of five methods on three real datasets, Jura and Concrete. The results were summarized by mean and standard deviation over 5 runs. Fig B. First two principle angles derived from the SLMM model for five real data. First principal angle is on left while the second principal angle is on right. Appendix E. Analysis between predictive performance and latent dimension size in ECoG dataset. Appendix F. Analysis between latent representation performance and latent dimension size in ECoG dataset. Fig C. Prediction performance on leave-one-channel-prediction task on different latent dimension size Q = 2, 4, 8 and 16. S1, S2, S3 and S4 represent four stimuli with paired of conditions (7627Hz, -10dB), (32000Hz, -10dB), (7627Hz, -50dB) and (32000Hz, -50dB). Fig D. Inferred orthonormalized latent functions from OSLMM and GPFA for all stimuli with Q = 10. (A-B) Eight stimuli for all attenuation with a fixed frequency 7627 Hz averaged by trials. (A) OSLMM; (B) GPFA); (C-D): The same type of inferred orthonormalized latent functions for OSLMM (C) and GPFA (D) but for all frequencies with a fixed attenuation -10 dB averaged by trials. Moreover, we conducted linear regression between the peak of latent functions and exogenous variable (attenuation or frequency). The R2 scores for OSLMM/GPFA are 0.71/0.61(Frequency: 7627) and 0.28/0.06(Attenuation: -10). Appendix G. [file pcbi.1011975.s001.pdf]

Supplementary Information: Bayesian inference of structured latent spaces from neural population activity with the orthogonal stochastic linear mixing model

Rui Meng[1] Kristofer E. Bouchard[1,2,3,4,*]

1 Biological Systems and Engineering Division, Lawrence Berkeley National Laboratory, Berkeley, CA, US
2 Scientific Data Division, Lawrence Berkeley National Laboratory, Berkeley, CA, US
3 Helen Wills Neuroscience Institute, University of California Berkeley, Berkeley, CA, US
4 Redwood Center for Theoretical Neuroscience, University of California Berkeley, Berkeley, CA, US

*corresponding author: kebouchard@lbl.gov

S1 File

Appendix A

Hyper-parameter learning for SLMM

When considering the independent noise such that Σ is a diagonal matrix, we set the a conjugate inverse Gamma prior $p(\Sigma) = \prod_{p=1}^{P} \mathcal{IG}(\sigma_p^2 | a, b)$, where σ_p^2 is the pth element on the diagonal of Σ. Then the conditional posterior distribution of σ_p^2 is

$$\begin{aligned}\sigma_p^2 | - &\propto \prod_{t=1}^{T} \mathcal{N}(\mathrm{y}_{tp} | \mathrm{g}_{tp}, \sigma_p^2) \mathcal{IG}(\sigma_p^2 | a, b) \\ &\sim \mathcal{IG}(\sigma_p^2 | a + \frac{N}{2}, b + \frac{\sum_{n=1}^{N} (\mathrm{y}_{tp} - \mathrm{g}_{tp})^2}{2}) \,. \end{aligned} \tag{1}$$

In practice, we set $a = 0.01$ and $b = 0.01$ to allow large variance.

We consider the commonly-used squared exponential (SE) covariance function for W and f

$$K_i(\mathrm{t}_1, \mathrm{t}_2) = \sigma_i^2 \exp(-\frac{\|\mathrm{t}_1 - \mathrm{t}_2\|^2}{2 l_i^2}) \tag{2}$$

where $i = W$ or f. $\sigma_f^2 = 1$ is fixed for model identifiability. We put a conjugate prior on σ_W^2 such that $\sigma_W^2 \sim \mathcal{IG}(c, d)$. Then the conditional posterior distribution is

$$\begin{aligned}\sigma_W^2 | - &\propto \prod_{p=1}^{P} \prod_{q=1}^{Q} \mathcal{N}(\mathbf{w}_{pq} | \mathbf{0}, \sigma_W^2 \tilde{\mathbf{K}}_w) \mathcal{IG}(\sigma_W^2 | c, d) \\ &\sim \mathcal{IG}(\sigma_W^2 | c + \frac{NPQ}{2}, d + \frac{\sum_{i=1}^{P} \sum_{j=1}^{Q} \mathbf{w}_{pq}' \tilde{\mathbf{K}}_W^{-1} \mathbf{w}_{pq}}{2}) \end{aligned} \tag{3}$$

where $\tilde{\mathbf{K}}_W$ is the correlation matrix and $\mathbf{K}_w = \sigma_W^2 \tilde{\mathbf{K}}_w$. As for length-scale parameters l_i^2, we put a non-informative prior $l_i^2 \propto \frac{1}{l_i^2}$ and sample them via adaptive Metropolis-with-Gibbs algorithm [1].

Appendix B

Theoretical proofs for sufficient statistics

Theorem $\mathbf{T}_n\mathbf{y}_n$ is a minimally sufficient statistic for $\mathbf{f}_n$.

Proof: Without loss of generality, we ignore the subscript n in this proof. To show $\mathbf{Ty}$ is a minimally sufficient statistic for $\mathbf{f}$, we need to prove $p(\mathbf{y}_1|\mathbf{f})/p(\mathbf{y}_2|\mathbf{f})$ is a constant as a function of $\mathbf{f}$ if and only if $\mathbf{Ty}_1 = \mathbf{Ty}_2$. We have

$$\begin{aligned}\log\frac{p(\mathbf{y}_1|\mathbf{f})}{p(\mathbf{y}_2|\mathbf{f})} &= \log\frac{\mathcal{N}(\mathbf{y}_1|\mathbf{U}\mathbf{S}^{\frac{1}{2}}\mathbf{f},\Sigma)}{\mathcal{N}(\mathbf{y}_2|\mathbf{U}\mathbf{S}^{\frac{1}{2}}\mathbf{f},\Sigma)}\\ &= (\mathbf{y}_1-\mathbf{y}_2)'\Sigma^{-1}\mathbf{U}\mathbf{S}^{\frac{1}{2}}\mathbf{f} + \text{const}\\ &= \mathbf{f}'\mathbf{S}^{\frac{1}{2}}\mathbf{U}'\Sigma^{-1}(\mathbf{y}_1-\mathbf{y}_2) + \text{const}\end{aligned}$$

When we consider the homogeneous noise $\Sigma = \sigma_y^2\mathbf{I}$, we have

$$\begin{aligned}\log\frac{p(\mathbf{y}_1|\mathbf{f})}{p(\mathbf{y}_2|\mathbf{f})} &= \frac{1}{\sigma_y^2}\mathbf{f}'\mathbf{S}^{\frac{1}{2}}\mathbf{U}'(\mathbf{y}_1-\mathbf{y}_2) + \text{const}\\ &= \frac{1}{\sigma_y^2}\mathbf{f}'\mathbf{S}^{\frac{1}{2}}\mathbf{U}'\mathbf{U}\mathbf{S}^{\frac{1}{2}}\mathbf{S}^{-\frac{1}{2}}\mathbf{U}'(\mathbf{y}_1-\mathbf{y}_2) + \text{const}\\ &= \mathbf{f}'\mathbf{S}^{\frac{1}{2}}\mathbf{U}'\Sigma^{-1}\mathbf{U}\mathbf{S}^{\frac{1}{2}}\mathbf{T}(\mathbf{y}_1-\mathbf{y}_2) + \text{const}\,. \end{aligned}\tag{4}$$

Because $\mathbf{S}^{\frac{1}{2}}\mathbf{U}'\Sigma^{-1}\mathbf{U}\mathbf{S}^{\frac{1}{2}}$ is invertible, Equation 4 does not depend on $\mathbf{f}$ if and only if $\mathbf{Ty}_1 = \mathbf{Ty}_2$. Therefore, $\mathbf{T}_n\mathbf{y}_n$ is a minimally sufficient statistic for $\mathbf{f}_n$.

Appendix C

Hyper-parameter learning for OSLMM

We consider the homogeneous noise such that $\Sigma = \sigma_y^2\mathbf{I}$ in this setting and we put a conjugate prior on the variance, $p(\sigma_y^2) = \mathcal{IG}(\sigma^2|a,b)$. The conditional posterior distribution is

$$\begin{aligned}\sigma_y^2|- &\propto \prod_{t=1}^{T}\mathcal{N}(\mathbf{y}_t|\mathbf{g}_t,\sigma_y^2\mathbf{I})\mathcal{IG}(\sigma_y^2|a,b)\\ &\sim \mathcal{IG}(\sigma_y^2|a+\frac{PT}{2}, b+\frac{\sum_{t=1}^{T}(\mathrm{y}_{td}-\mathrm{g}_{td})^2}{2})\,.\end{aligned}\tag{5}$$

We consider the commonly-used SE covariance function for $\boldsymbol{h}$ and $\boldsymbol{f}$. $\sigma_f^2 = 1$ is fixed for model identifiability. We put a conjugate prior on σ_h^2 such that $\sigma_h^2 \sim \mathcal{IG}(c,d)$. Then the conditional posterior distribution is

$$\begin{aligned}\sigma_h^2|- &\propto \prod_{q=1}^{Q}\mathcal{N}(\mathbf{h}_q|\mathbf{0},\sigma_h^2\tilde{\mathbf{K}}_h)\mathcal{IG}(\sigma_h^2|c,d)\\ &\sim \mathcal{IG}(\sigma_W^2|c+\frac{QT}{2}, d+\frac{\sum_{q=1}^{Q}\mathbf{h}_q'\tilde{\mathbf{K}}_h^{-1}\mathbf{h}_q}{2})\end{aligned}\tag{6}$$

where $\tilde{\mathbf{K}}_h$ is the correlation matrix and $\mathbf{K}_h = \sigma_h^2\tilde{\mathbf{K}}_h$. The corresponding length-scale parameters learninng is the same as that for SLMM.

Appendix D

Prediction comparison on real datasets

We compared SLMM and OSLMM to GPRN models with the following inference approaches: (1) MFVB – mean-field variational Bayes inference [2], (2) NPV – nonparametric variational Bayes inference [3], (3)SGPRN – scalable variational Bayesian inference [4]. For both SLMM and OSLMM, Markov Chain Monte Carlo had 500 iterations, in which the first 200 iterations are used for burnin. For the variational methods, GPRN(MFVB) and GPRN(NPV) ran 100 iterations and SGPRN ran 2000 epochs to ensure convergence.

We evaluated the model performances on five real-world datasets, **Jura**, **Concrete**, **Equity**, **PM2.5** and **Neural**, with $3, 3, 25, 100$ and 128 outputs respectively. Specifically, (1) **Jura**, the concentrations of cadmium at 100 locations within a 14.5 km^2 region in Swiss Jura. Following [4], we utilized the concentrations of cadmium, nickel, and zinc at 259 nearby locations to predict the three correlated concentrations at another 100 locations. (2) **Concrete**, a geostatistics dataset, including 103 samples with 7 concrete mixing ingredients as input variables and with 3 output variables (slump, flow, and compressive strength). We random split it into a training set of 80 points and a test set of 23 points as in [3]. (3)**Equity**, a financial dataset consists of 643 records of 5 equity indices. The task is to predict the 25 pairwise correlations. Following [2] we randomly chose 200 records for training and chose another 200 records for testing. (4) **PM2.5**, 100 spatial measurements of the particulate mater pollution (PM2.5) in Salt Lake City in July 4-7, 2018, where inputs are time stamps. We randomly took 256 samples for training and 32 for testing. (5) **Neural**, a micro-electrocorticography (µm ECoG) recordings from rat auditory cortex in response to pure tone pips collected in the Bouchard Lab [5].We randomly selected 100 samples for training and another 100 for testing. For all datasets, we normalized each input dimension to have zero mean and unit variance; for **Jura**, **Concrete** and **Neural** data, the outputs in each dimension are normalized to have zero mean and unit variance.

We report the predictive mean absolute error for datasets with moderate-to-large output dimension **Equilty**, **PM2.5** and **Neural** in Table A. For datasets with small output dimension (**Jura** and **Concrete**), the predictive performance of OSLMM does not significantly outperform other methods, and gives similar results to GPRN(NPV). This may be because the output correlation is trivial. We provide the predictive mean absolute error for those two datasets in Appendix A. All results were summarized by the mean and standard deviation over 5 runs with latent dimension $Q = 2$. Table A shows that the prediction performance of OSLMM is uniformly and robustly better than the other four methods.

Table A. Predictive mean absolution error of five methods on three real datasets, **Equilty**, **PM2.5** and **Neural**. The results were summarized by mean and standard deviation over 5 runs.

	**Equity**	**PM2.5**	**Neural**
SLMM	2.6995e-5 (7.6614e-7)	9.5514 (0.3703)	0.6068 (0.0018)
OSLMM	**2.6643e-5** (2.5686e-7)	**3.9699** (0.2595)	**0.5141** (0.0206)
GPRN (MFVB)	3.0327e-5 (8.1183e-7)	5.9738 (1.3893)	0.5654 (0.0047)
GPRN (NPV)	4.3490e-5 (5.9300e-6)	6.1794 (1.4397)	0.5724 (0.0051)
SGPRN	2.7346e-5 (1.4374e-7)	8.6163 (2.1070)	0.5727 (0.0263)

Next, we compared SLMM, OSLMM and SGPRN in terms of compute speed, since GPRN(MFVB) and GPRN(NPV) are known to be very slow [4]. We report the per-iteration running time of SLMM and OSLMM, and the average time of 4 epochs

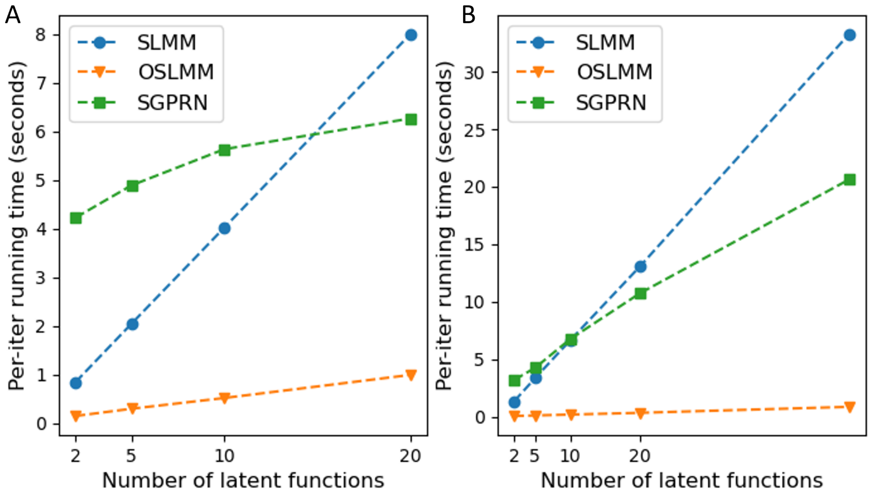

Fig A. Training speed of SLMM, OSLMM and SGPRN inference algorithms on **Equity** data (A) and **PM2.5** data (B). We show the running time per iteration in the setting with different number of latent functions.

of SGPRN for a fair comparison. For all three methods, because the number of latent functions Q should be smaller than output dimension, $Q < P$, we varied the size of the latent functions, $Q = (2, 5, 10, 20, 50)$ for **PM2.5** and **Neural** and the size $Q = (2, 5, 10, 20)$ for **Equity**. We report the result of **Neural** in Fig **??** and the results of **Equity** and **PM2.5** in Fig A.These results clearly demonstrate that inference of OSLMM faster than SLMM and SGPRN.

On the other hand, we reported the predictive mean absolution error of five methods on two real datasets, **Jura** and **Concrete** in Table B

Table B. Predictive mean absolution error of five methods on three real datasets, **Jura** and **Concrete**. The results were summarized by mean and standard deviation over 5 runs.

	**Jura**	**Concrete**
SLMM	0.6643 (0.0103)	0.7627 (0.0507)
OSLMM	0.6230 (0.0079)	**0.5305** (0.0245)
GPRN (MFVB)	0.6346 (0.0047)	0.7145 (0.1560)
GPRN (NPV)	**0.6218** (0.0113)	0.5567 (0.0225)
SGPRN	0.6762 (0.0669)	0.8331 (0.0199)

Evaluation of assumption of fixed embedding subspace

In contrast to SLMM, OSLMM uses a fixed embedding space. To evaluate the assumption of a fixed subspace in real data, we determined the variability of embedded space in SLMM by calculating and plotting the principal angles. The principal angles are defined between span$[W(t)]$ and span$[\tilde{W}]$ in which the optimized space minimizes the sum of the cosine distance between the optimal space and embedding space. Mathematically the basis of the optimal space is defined as $\tilde{W} = \min_W(\sum_t \cos\theta_{W,W(t)})$. We plot the distributions of principal angles for five real data in Fig B. We found that there could be considerable variations of subspaces in the real data. However, the prediction performance in **Table S1** and **Table S2** demonstrated that SLMM performs substantially worse than OSLMM. This implies

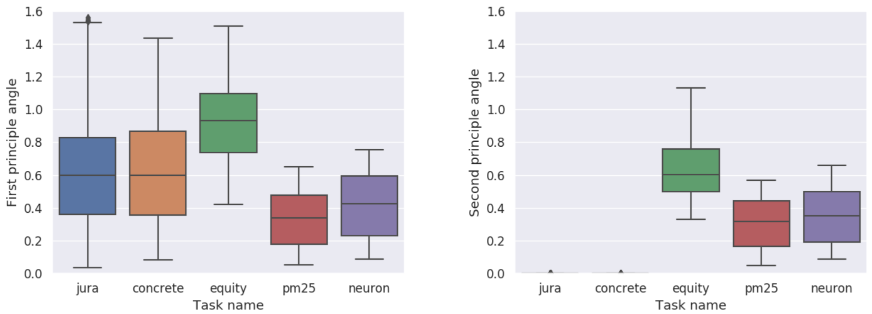

Fig B. First two principle angles derived from the SLMM model for five real data. First principal angle is on left while the second principal angle is on right.

that SLMM is too flexible, and is over-fitting the data. As such, putting fixed embedding assumptions seems to help model generalization, as was done in OSLMM. Note that although OSLMM assumes a fixed latent embedding subspace, the coefficient function is modeled more flexibly which gets rid of the Gaussian assumption. We have illustrated the benefits of the flexible modeling by comparing it with the GPFA model in different settings in the main text. In summary, OSLMM balances computational efficiency with model flexibility, and is applicable to the non-Gaussian case.

Appendix E

Analysis between predictive performance and latent dimension size in ECoG dataset

We conduct leave-one-channel-prediction tasks on the ECoG data for the same four stimuli S1, S2, S3 and S4 with different latent dimension $Q = 2, 4, 8$ and 16. We provide the prediction error and R^2 in Fig C. It shows that for most of channels and most of selection of Q, OSLMM outperforms GPFA in predictive performance. And we also find that when $Q > 2$, OSLMM outperforms GPFA for all four stimuli.

Appendix F

Analysis between latent representation performance and latent dimension size in ECoG dataset

We explore the relation between latent representation performance and latent dimension size by conducting OSLMM and GPFA on the ECoG data for all trials. We exploit different latent representation under different latent dimension size $Q = 5, 10$ and 15. We display the first three principle components in the latent space in Fig **??**, Fig D and Fig E. Those figures show that the latent representations of first three principle components have robust superior representations across different latent dimensions Qs.

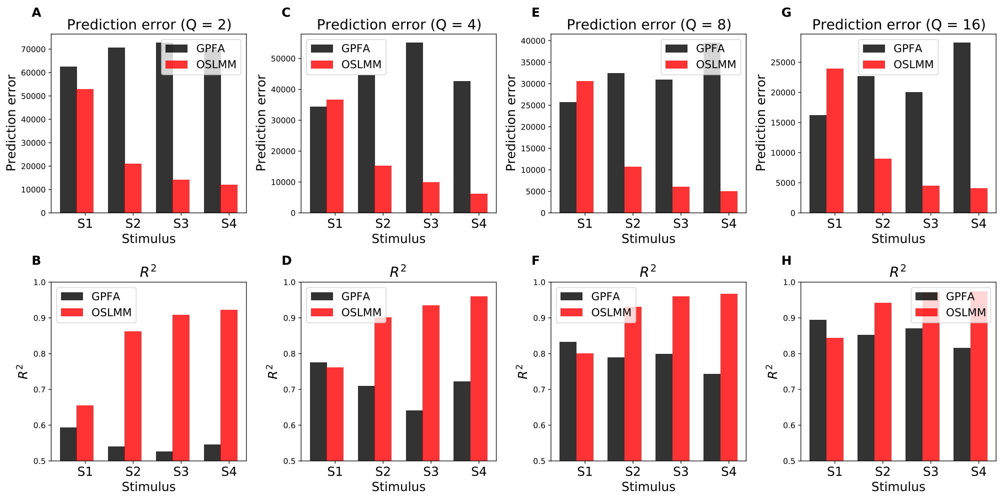

Fig C. Prediction performance on leave-one-channel-prediction task on different latent dimension size $Q = 2, 4, 8$ and 16. S1, S2, S3 and S4 represent four stimuli with paired of conditions (7627Hz, -10dB), (32000Hz, -10dB), (7627Hz, -50dB) and (32000Hz, -50dB).

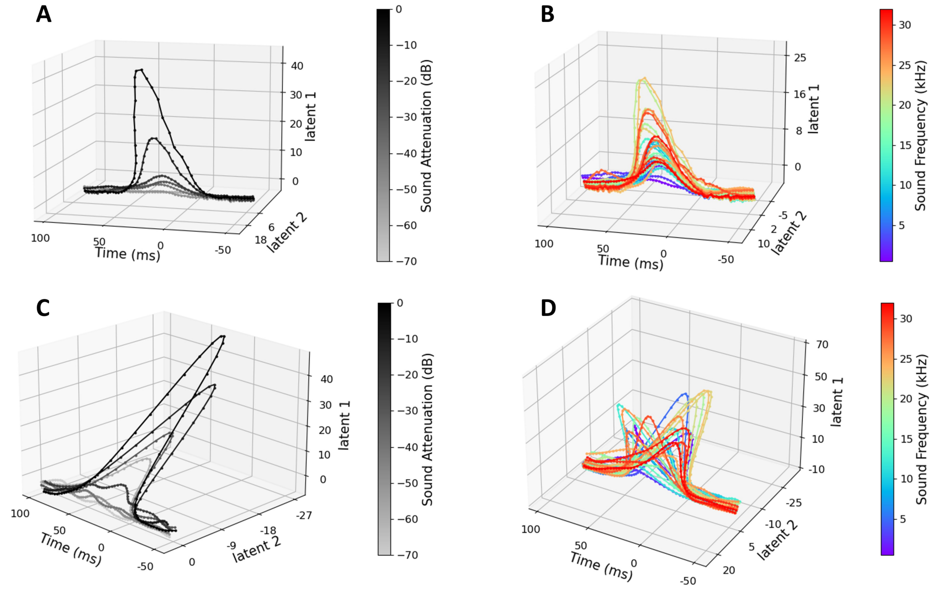

Fig D. Inferred orthonormalized latent functions from OSLMM and GPFA for all stimuli with $Q = 10$.(A-B) Eight stimuli for all attenuation with a fixed frequency 7627 Hz averaged by trials. (A) OSLMM; (B) GPFA); (C-D):The same type of inferred orthonormalized latent functions for OSLMM (C) and GPFA (D) but for all frequencies with a fixed attenuation -10 dB averaged by trials. Moreover, we conducted linear regression between the peak of latent functions and exogenous variable (attenuation or frequency). The R^2 scores for OSLMM/GPFA are 0.71/0.61(Frequency: 7627) and 0.28/0.06(Attenuation: -10).

Appendix G

Latent trajectories with/without scaling

GPFA models the output $y(t)$ using $Wf(t)$. This models the temporal structure of $y(t)$ as $cov(y) = WSW^T$ where $S = cov(f)$. This (linear) approach implies a separable

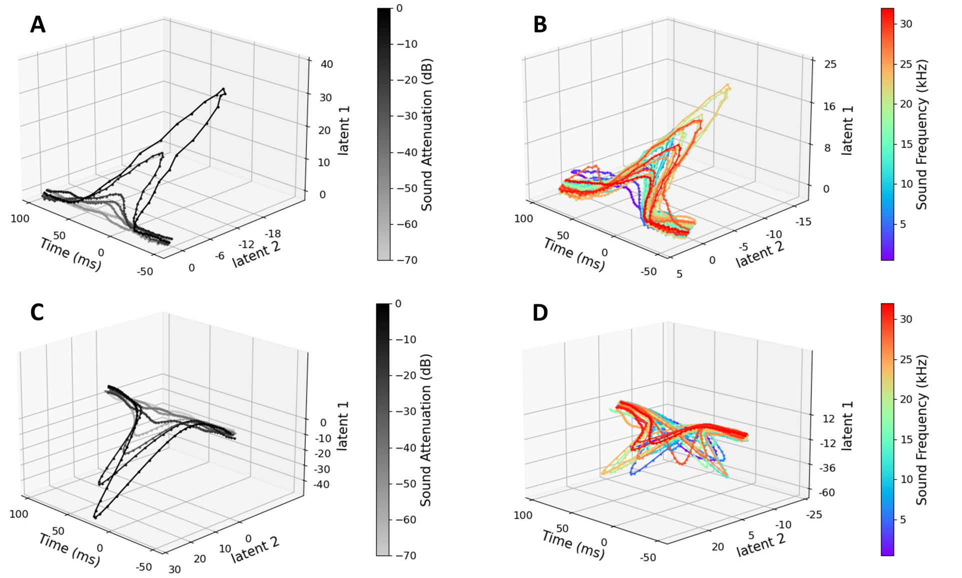

Fig E. Inferred orthonormalized latent functions from OSLMM and GPFA for all stimuli with $Q = 15$.(A-B)Eight stimuli for all attenuation with a fixed frequency 7627 Hz averaged by trials. (A) OSLMM; (B) GPFA); (C-D):The same type of inferred orthonormalized latent functions for OSLMM (C) and GPFA (D) but for all frequencies with a fixed attenuation -10 dB averaged by trials. Moreover, we conducted linear regression between the peak of latent functions and exogenous variable (attenuation or frequency). The R^2 scores for OSLMM/GPFA are 0.85/0.62(Frequency: 7627) and 0.50/0.06(Attenuation: -10).

model in which the correlation function is restricted to be the product of correlation functions from parameter space (i.e., W) and time domain (i.e., $f(t)$), respectively. Thus, GPFA cannot capture relationships between parameters and time. In OSLMM, we model $y(t) = W(t)f(t)$, which flexibly handles the cross-correlation through the product of the time-varying matrix $W(t)$ and the time-varying function $f(t)$. Further, because both $W(t)$ and $f(t)$ have Gaussian distributions, OSLMM extends the Gaussian data assumption of GPFA to the non-Gaussian case, since the product of two Gaussians is strictly non-Gaussians. Both GPFA and OSLMM are not identifiable. In other words, $y(t) = W(t)f(t) = W(t)f(t)$ where $W(t) = W(t)P, f(t) = P^{-1}f(t)$ where P is a perturbation matrix. But the embedding subspace of $y(t)$, $span(W(t))$, is identifiable for both GPFA and OSLMM. Hence, in the main text, we focus our analysis on that.

To gain intuition into the role of W(t) in the observed trajectories, we first visualized the latent neural trajectories of the ECoG auditory responses in Fig **??** with (**A,C**) and without (**B,D**) the time varying scale factor. Visually, we observed that the differences were entirely in the magnitude of projection, and the geometry of the trajectories with respect to each other and their relationship to the stimulus parameters (attenuation, top; frequency, bottom), were essentially unaltered. We also computed the log scales of latent trials $h_{q(t)}$ described in the OSLMM section, and plotted the smoothed trials (with rolling average with window size 7). This shows that, in this case, the log scale of latent trajectories can also match the dynamics of the stimulus evoked activity, with a loose ordering of log scale magnitude across dimensions (colors in **E**).

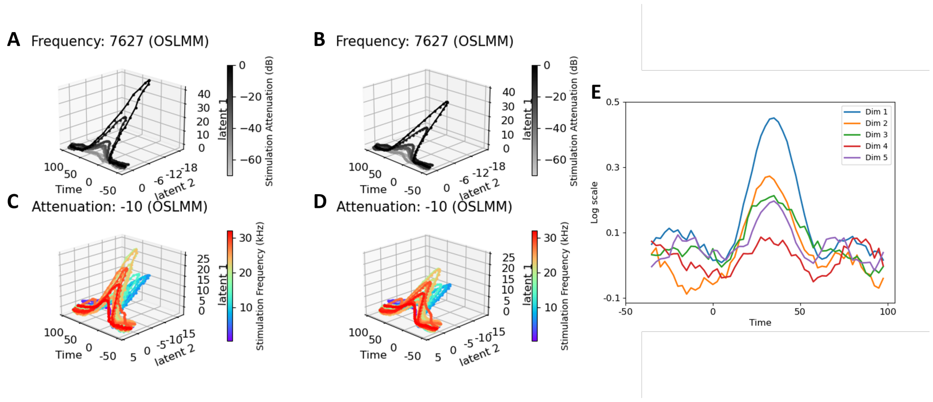

Fig F. Latent trajectories of ECoG auditory responses with (A and C) and without (B and D) the time varying scale factor. The log scale trajectories of ECoG auditory responses ranked by the corresponding variance (E).

For the motor cortex data, we performed the same analysis in Fig G. On in **A** is the unscaled neural trajectories color coded by reach angle, while in **B** is the scaled version. We also computed the log scales of latent trials $h_{q(t)}$ described in the OSLMM section, and plotted the smoothed trials (**C**, with rolling average with window size 7). In contrast to the auditory cortex trajectories, the geometry of latent neural trajectories were substantially different between the two. In particular, the unscaled trajectories (**A**) were much more tangled and had less organization with respect to the reach angle compared to the scaled trajectories (**B**).

Together, the results in **Figs S6 and S7** indicate that there does not appear to be a consistent or easily understandable impact of W(t) on the latent neural trajectories across these data sets. Specifically, as is demonstrated by the analysis of the auditory cortex data, compared to GPFA, OSLMM latent neural trajectories can be substantially more structured by external parameters even without the inclusion of W(t), suggesting that the orthogonality constraints is also playing an important role. However, the results for the motor cortex were harder to interpret.

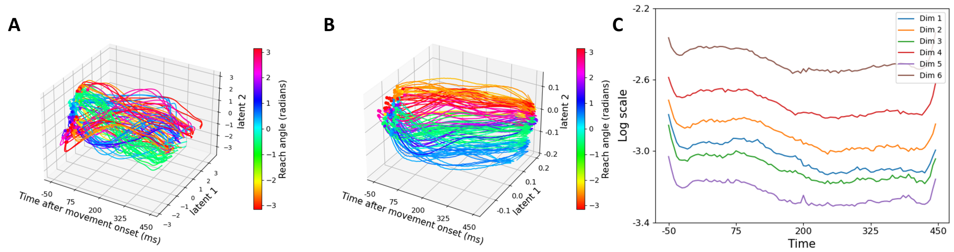

Fig G. Time varying scale analysis in motor cortex data. Latent trajectories with (A) and without (B) time-varying scale. The log scale trajectories of motor cortex responses ranked by the corresponding variance (C)

Distance plots for latent trajectories.

We further quantified the dynamics of structure in the latent spaces by measuring the point-wise distance between individual trajectory and baseline trajectory. For the analysis of reach angle, the baseline trajectory is the defined by the point-wise average of trajectory whose angle is within 0.5 radians. And for the analysis of speed, the

baseline trajectory is defined as the trajectory with the slowest speed. We provided the plots in Fig H.

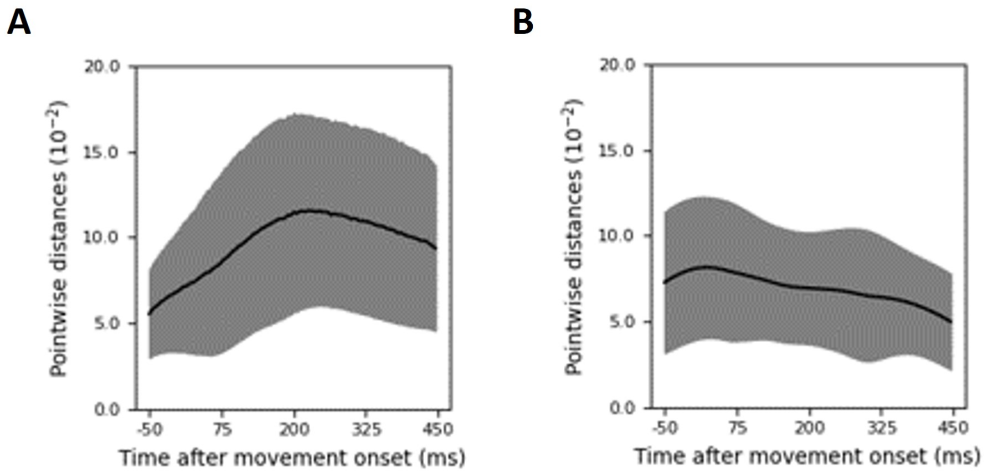

Fig H. Distance plots of latent trajectories for OSLMM (A) and GPFA (B). The mean and one standard deviation below and above it for the point-wise distances are provided.

References

1. Roberts GO, Rosenthal JS. Examples of adaptive MCMC. Journal of Computational and Graphical Statistics. 2009;18(2):349–367.
2. Wilson AG, Knowles DA, Ghahramani Z. Gaussian process regression networks. arXiv preprint arXiv:11104411. 2011;.
3. Nguyen T, Bonilla E. Efficient variational inference for Gaussian process regression networks. In: Artificial Intelligence and Statistics. PMLR; 2013. p. 472–480.
4. Li SL, Xing W, Kirby RM, Zhe S. Scalable Gaussian Process Regression Networks. In: International Joint Conference on Artificial Intelligence-Pacific Rim International Conference on Artificial Intelligence (IJCAI-PRICAI); 2020. p. 2456–2462.
5. Dougherty ME, Nguyen AP, Baratham VL, Bouchard KE. Laminar origin of evoked ECoG high-gamma activity. In: 2019 41st Annual International Conference of the IEEE Engineering in Medicine and Biology Society (EMBC). IEEE; 2019. p. 4391–4394.
